# Genetic and molecular dissection of ginseng (*Panax ginseng* Mey.) germplasm using high-density genic SNP markers, secondary metabolites, and gene expressions

**DOI:** 10.3389/fpls.2023.1165349

**Published:** 2023-07-28

**Authors:** Sizhang Liu, Yue Jiang, Yanfang Wang, Huimin Huo, Mustafa Cilkiz, Ping Chen, Yilai Han, Li Li, Kangyu Wang, Mingzhu Zhao, Lei Zhu, Jun Lei, Yi Wang, Meiping Zhang

**Affiliations:** ^1^ College of Life Science, Jilin Agricultural University, Changchun, Jilin, China; ^2^ College of Chinese Medicinal Materials, Jilin Agricultural University, Changchun, Jilin, China; ^3^ Department of Soil and Crop Sciences, Texas A&M University, College Station, TX, United States; ^4^ Research Center for Ginseng Genetic Resources Development and Utilization, Jilin Province, Jilin Agricultural University, Changchun, Jilin, China

**Keywords:** ginsenoside, population structure, genetic diversity, phylogeny, ginsenoside biosynthesis

## Abstract

Genetic and molecular knowledge of a species is crucial to its gene discovery and enhanced breeding. Here, we report the genetic and molecular dissection of ginseng, an important herb for healthy food and medicine. A mini-core collection consisting of 344 cultivars and landraces was developed for ginseng that represents the genetic variation of ginseng existing in its origin and diversity center. We sequenced the transcriptomes of all 344 cultivars and landraces; identified over 1.5 million genic SNPs, thereby revealing the genic diversity of ginseng; and analyzed them with 26,600 high-quality genic SNPs or a selection of them. Ginseng had a wide molecular diversity and was clustered into three subpopulations. Analysis of 16 ginsenosides, the major bioactive components for healthy food and medicine, showed that ginseng had a wide variation in the contents of all 16 ginsenosides and an extensive correlation of their contents, suggesting that they are synthesized through a single or multiple correlated pathways. Furthermore, we pair-wisely examined the relationships between the cultivars and landraces, revealing their relationships in gene expression, gene variation, and ginsenoside biosynthesis. These results provide new knowledge and new genetic and genic resources for advanced research and breeding of ginseng and related species.

## Introduction

Ginseng, including Asian ginseng (*Panax ginseng* Mey.) and American ginseng (*P. quinquefolius* L.), has long been used for healthy food and medicine (Ichim and de Boer, 2021; Barido et al., 2022). As healthy food supplements, ginseng has been used in a variety of ginseng food products, such as beverage, energy drinks, liquor (Wang et al., 2019; Yu and Yue, 2021), candy, tea, jam, chocolate (Chung et al., 2011), and healthy food (Barido et al., 2022). As the expectation of people for health increases, more varieties of ginseng food products will be produced and consumed worldwide. As a medicinal herb, ginseng has numerous effects on human health. These include, but are not limited to, preventing cardiovascular diseases (Wan et al., 2023), relieving pain (Choi, 2008), improving brain function (Liu et al., 2023), increasing antitumor ability (Fan et al., 2023), providing energy boost (Luo and Huang, 2023), lowering blood sugar and cholesterol levels (Jin et al., 2019), reducing stress and fatigue (Tardy et al., 2021), treating diabetes (Li et al., 2023) and man’s sexual dysfunction (Farnia et al., 2019), modulating the immune system (You et al., 2022), and slowing aging (de Oliveira Zanuso et al., 2022). In addition, ginseng has been a desirable species for studies of the molecular mechanisms underlying the biosynthesis of triterpene secondary metabolites in plants (Kim et al., 2009; Kim et al., 2014; Kim et al., 2018).

Studies have documented that ginseng has a number of bioactive ingredients for human health, of which the most recognized are ginsenosides (Choi, 2008; You et al., 2022; Fan et al., 2023; Wan et al., 2023), which are secondary metabolites—a class of triterpenoid saponin glycosides. Ginsenosides have been studied extensively, not only in medical functions for human health but also in biochemistry and molecular biology. At least 70 ginsenosides have been identified in ginseng (Ru et al., 2015), and it has been found that the content of each ginsenoside varied across genotypes, development stages, and plant parts and influenced by environments (Lee et al., 2017; Xiu et al., 2019; Jiang et al., 2022). Genetic studies showed that the content of each ginsenoside is inherited as a quantitative trait and, therefore, likely controlled by multiple genes (Zhang et al., 2020b; Jiang et al., 2022). So far, 11 genes that encode the key enzymes involved in ginseng ginsenoside biosynthesis have been cloned, including beta-amyrin synthase (*β-AS*) (Kushiro et al., 1998), cycloartenol synthase (*CAS*) (Kim et al., 2009), dammarenediol synthase (*DS*) (Han et al., 2006), farnesyl pyrophosphate synthase (*FPS*) (Kim et al., 2014), squalene epoxidase (*SE*) (Han et al., 2020), squalene synthase (*SS*) (Lee et al., 2004; Kim et al., 2011), UDP-glycosyltransferase (*UGT71A27*) (Jung et al., 2014), UDP-glycosyltransferase (*UGT74AE2*) (Jung et al., 2014), cytochrome P450 (*CYP716A53v2*) (Han et al., 2012), cytochrome P450 (*CYP716A52v2*) (Han et al., 2013), and cytochrome P450 (*CYP716A47*) (Han et al., 2011). Moreover, transcriptome-wide gene expressions have been profiled from different genotypes of ginseng (Li et al., 2021), different developmental stages (Jayakodi et al., 2015; Wang et al., 2015), different plant parts (Li et al., 2013; Wang et al., 2015), and different treatment conditions (Cao et al., 2015) to identify candidate genes involved in the ginsenoside biosynthesis. Nevertheless, neither ginsenoside contents nor gene expressions have been used to molecularly characterize ginseng germplasm.

The germplasm collection of a species is the gene pool of the species containing most, if not all, of the genes necessary for its genetic improvement and adaptation to climate change. Therefore, genetic and molecular evaluation and dissection of a species’ germplasm collection is paramount to its efficient utilization for continued genetic improvement, gene discovery, and favorable allele mining and for deciphering the species’ speciation, genetic variation, and evolution (Jia et al., 2017; Zhang et al., 2021b). Several types of DNA markers have been used to decipher the molecular diversity and population structure of a species using a representative selection of its germplasm lines. These DNA markers include randomly amplified polymorphic DNAs (RAPDs) (Wang et al., 2016), simple sequence repeats (SSRs) (An et al., 2017; Lee et al., 2020a; Lee et al., 2020b), and single-nucleotide polymorphisms (SNPs) generated by GBS (genotyping-by-sequencing) with ddRAD-seq (double-digested restriction site-associated DNA sequencing) (Song et al., 2021), whole-genome resequencing (Fang et al., 2017; Ma et al., 2018), or RNA-seq (RNA sequencing) (Liang and Schnable, 2016; Jehl et al., 2021; Thorstensen et al., 2021). In ginseng, Wang et al. (2016) analyzed its genetic diversity and population structure with 44 samples collected from Heilongjiang, Liaoning, and Jilin Provinces of China as well as the People’s Republic of Korea using 41 RAPD markers. An et al. (2017) studied the genetic diversity and population structure of Chinese ginseng with 73 accessions collected from Jilin, China, using eight SSR markers. Lee et al. (2020a) studied the molecular diversity and population structure of ginseng germplasm with 1,109 accessions collected from South Korea (899), China (202), Japan (4), USA (3), and Russia (1) using 17 SSR markers. Lee et al. (2020b) studied the genetic composition of Korean ginseng germplasm by collection area and resource type with 451 accessions using 33 SSR markers. Nevertheless, these studies used only a smaller number of accessions (Wang et al., 2015; An et al., 2017), or 8 to 41 DNA markers (Wang et al., 2015; An et al., 2017; Lee et al., 2020a, Lee et al., 2020b). Use of the smaller number of accessions or smaller number of markers limited comprehensive understanding of the genetic diversity and population structure of ginseng. The germplasm research of Chinese ginseng, especially at the genomic level, is limited, thus restricting its utilization for ginseng breeding and gene discovery. Jilin, China, where over 56% of the world’s ginseng is produced (Baeg and So, 2013; Chen et al., 2022), has been considered one of the ginseng origin and diversity centers, which is known as Jilin ginseng. However, comprehensive analysis of Jilin ginseng germplasm remains.

The aims of the present study were to construct a mini-core germplasm collection representative for Jilin ginseng and characterize the collection at genetic and molecular levels. We first collected 1,168 germplasm lines from the origin and diversity center of Jilin ginseng, developed the mini-core germplasm collection, and analyzed its genetic variation using 16 ginsenosides. Then, we sequenced the 4-year-old plant root transcriptomes of the mini-core collection, identified the SNPs contained in functional genes, quantified the expressions of the genes and transcripts, and characterized it using high-density genic SNP markers and gene expression profiles to facilitate gene discovery, favorable allele mining, and application of the research result for enhanced breeding. Analysis of the genic SNPs and gene expressions provided a comprehensive insight into molecular diversity, population structure, and evolution of the ginseng species. In addition, a relationship of the ginsenosides was proposed in the biosynthesis of ginsenosides. These resources and findings promote ginseng research such as genome-wide identification of genes involved in ginsenoside biosynthesis and enhance ginseng breeding such as marker-assisted selection and gene-based breeding (Liu et al., 2020; Zhang et al., 2020a; Zhang et al., 2021a; Liu et al., 2022a; Liu et al., 2022b; Zhang et al., 2022).

## Materials and methods

### Construction of a mini-core germplasm collection

**Figure 1:**
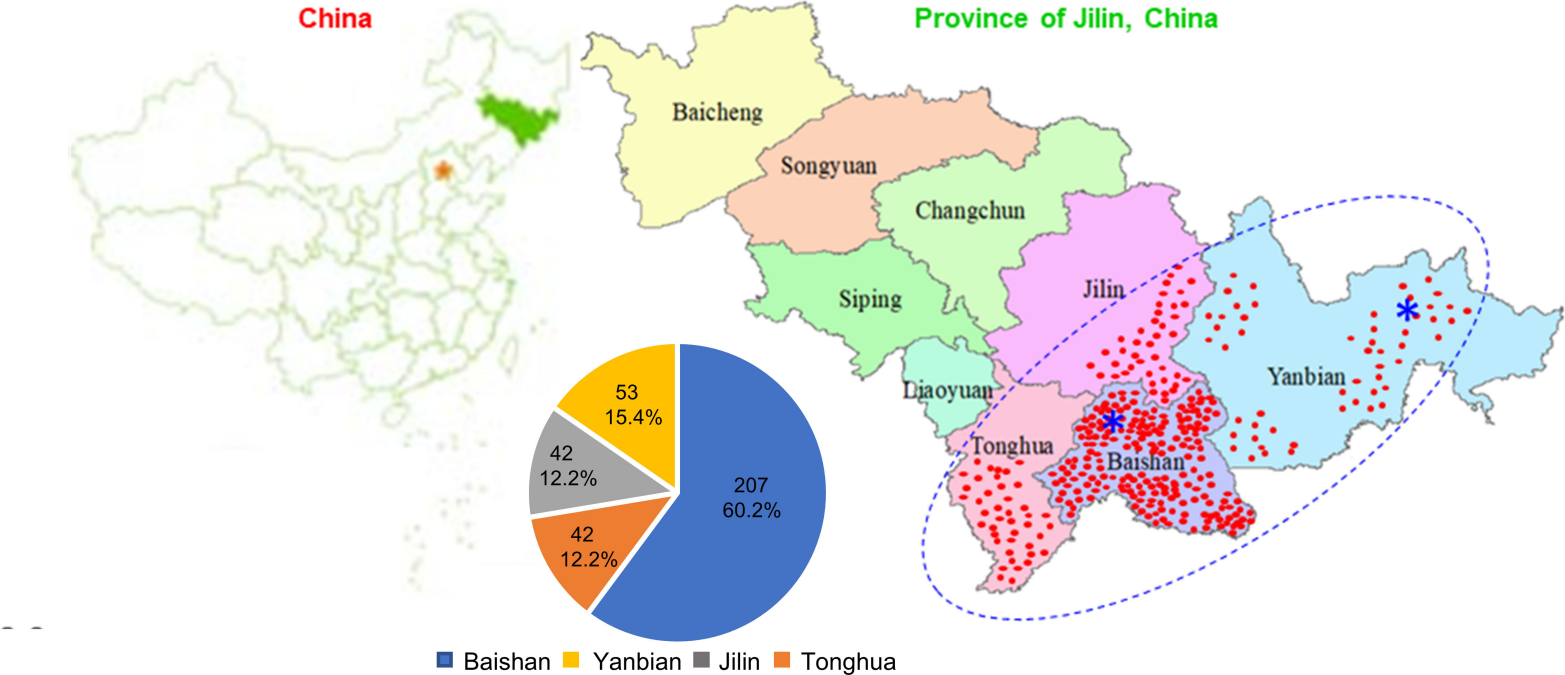
Geographical distribution and constitution of the Jilin ginseng mini-core collection. The collection consists of 344 cultivars and landraces originated from Jilin Province (43°42'N 126°12'E), China. The red dots indicate the locations where the cultivars and landraces were collected. Blue asterisks (*) indicate the Ginseng Research Experimental Stations, Jingyu County, Baishan and Wangqing County, Yanbian, where the samples for ginsenoside measurement and RNA sequencing were collected. The blue dot oval indicates the origin and diversity center of Jilin ginseng. For details of their origins, see **Table S1**.

A mini-core germplasm collection that is representative for the genetic variation and diversity of traits and genes in a species is necessary for its deeper research in genetics, genomics, and breeding. Therefore, to develop a mini-core germplasm collection for Jilin ginseng, we previously collected a total of 1,168 cultivars and landraces from 16 major ginseng-producing counties of Baishan, Jilin, Tonghua, and Yanbian, Jilin Province (43°42'N 126°12'E), China, in 2009 (**Figure 1**). The seeds of the cultivars and landraces were stratified to break their dormancy and planted at two Ginseng Research Experimental Stations, Jingyu, Baishan, and Wangqing, Yanbian, in 2010 (**Figure 1**). The experiments followed randomized complete block design, with two replicates, a distance of 15 cm between rows and a distance of 10 cm between plants. The management practice followed those locally used for ginseng production (Cui et al., 2010). In 2014, the 4-year-old plant roots of the 1,168 cultivars and landraces were harvested from five random plants per cultivar or landrace on 20/09/2014, frozen in liquid nitrogen, and stored in -80°C freezers for further analysis.

During plant growth and development from 2010 to 2014, the cultivars and landraces were recorded for main quality and yield potential traits and phylogenetically analyzed with 50 SSR markers (Yang, 2010; Luan, 2012). The quality and yield potential traits included plant height, stem color, days to first flower, number of flowers per plant, and number of fruits per plant. When harvested in 2014, recorded were shape of root, length of main root, diameter of main root, and weight of root. A phylogenetic tree of the cultivars and landraces was constructed based on their genotypes of the 50 SSR markers (Yang, 2010; Luan, 2012) using the PAUP software (Swofford, 2001). The cultivars and landraces of the mini-core collection were selected from the 1,168 cultivars and landraces, based on their morphological traits, their positions in the SSR phylogenetic tree, their geographical distribution, production in Jilin, China, and their representativeness for Jilin ginseng.

### Transcriptome sequencing

The root sample collected above from one root per cultivar or landrace was used for transcriptome sequencing. Zhang et al. (2019) showed that the expressions of gene transcripts quantified by RNA-seq were highly reproducible, varying from correlation coefficient *r* = 0.90 to 0.98 (*P* < 0.0001) between plants collected from a field trial replicate, different field trial replicates, and across years. Therefore, the mRNA of one plant root was sequenced for this study. Total RNA was extracted using the RNeasy Plant Mini Kit (Qiagen, Germany), following the manufacturer’s protocol. The integrity and quantity of the RNAs were estimated by Agilent 2100 Bioanalyzer (Agilent Technologies, USA). Poly(A) mRNA was purified from total RNA by NEBNext Oligo(dT)_25_ beads (NEB, USA). cDNA libraries were constructed from the mRNAs using the NEBNext Ultra RNA Library Prep Kit for Illumina (NEB, USA), as described by the manufacturer. Library sequencing was accomplished using the Illumina HiSeq 4000 platform with a 150-base paired-end (150PE) module at Gene Denovo Biotechnology Co (Beijing, China). Raw reads of low quality were removed, including those with adapters, containing more than 10% unknown nucleotides, and containing more than 50% low-quality bases (<Q10).

### SNP calling and genotyping

Single-nucleotide polymorphisms (SNPs) and nucleotide insertions/deletions (InDels) were called and genotyped among the mini-core collection cultivars and landraces by the SAMtools software with the default parameters (Li et al., 2009) using the ginseng genome sequence (Kim et al., 2018) as the reference. The SNPs that had a missing data frequency of >10%, a minor allele frequency (MAF) of <10%, and a base calling quality of <Q30 were filtered out. The remaining high-quality SNPs that had a missing data frequency of ≤10%, a MAF of ≥10%, and a base calling quality of ≥Q30 were used for further analysis.

### Population structure analysis

We constructed the population structure of the mini-core collection using STRUCTURE 2.3.4 (Pritchard et al., 2000; Porras-Hurtado et al., 2013). Because extremely extensive high-performing computing time is needed if all high-quality SNPs were used, a representative selection of high-quality SNPs was selected, with one SNP in approximately every 500 kb along the ginseng genome, for the population structure analysis. It was tested with selections of 1,000 and 6,232 of the SNPs that the reduced numbers of SNPs allowed consistent construction of the population structure of the mini-core collection (**Figure S1**). The Bayesian Markov Chain Monte Carlo (MCMC) model was used to estimate the population structure of the mini-core collection, with three runs for each number of subpopulations (K) from 2 to 10. Both burn-in time and MCMC replication number were set to 100,000 for each run. The optimal number of subpopulations for the mini-core collection was determined based on the LnP(K) and ΔK methods, respectively, and selected according to the results from both methods. When LnP(K) was approaching its maximum value and ΔK had a peak, the K value corresponded to the optimal number of subpopulations of the population under study (Evanno et al., 2005). The Q matrix was constructed based on the optimal K value. If a cultivar or landrace of the mini-core collection had 70% (0.70) or more of the genetic material that originated from an ancestry (hereafter defined ancestry index, with 1.00 for a cultivar solely originated from that ancestry), it was assigned to the subpopulation that had origin of that ancestry. However, if a cultivar or landrace had an ancestry index of less than 70% (0.70), it was considered to originate from this ancestry and one or more other ancestries and assigned to the admixture group.

Furthermore, the population structure of the mini-core collection was confirmed by phylogenetic analysis, followed by principal component analysis (PCA) using all high-quality genic SNPs. The phylogenetic tree was constructed using the TASSEL 5.2.79 software (Bradbury et al., 2007) with the neighbor-joining (NJ) method and 1,000 bootstrap replications. The genetic distances between cultivars or landraces calculated for their phylogenetic analysis were further pairwise analyzed between cultivars or landraces of the mini-core collection that were grouped into subpopulations. Because the cultivars and landraces of the admixture group were derived from introgression of the cultivars and landraces between two or three subpopulations of the mini-core collection, they were excluded from the genetic distance analysis. PCA was also carried out using the TASSEL 5.2.79 software with the default parameters (Bradbury et al., 2007).

### Molecular diversity analysis

The molecular diversity of the mini-core collection was estimated using allele frequency, gene diversity, heterozygosity, and polymorphism information content (PIC). The allele frequency, gene diversity, heterozygosity, and PIC were determined with the PowerMarker software (Liu and Muse, 2005) using the above selection of high-quality genic SNPs used for the population structure analysis of the mini-core collection.

### Extraction and content quantification of ginsenosides

Because ginsenosides are the main bioactive components for healthy food and medicine, we assayed the contents of ginsenosides in the roots of the cultivars and landraces of the mini-core collection to further characterize it. The roots of the cultivars and landraces collected above, with two roots per cultivar or landrace and one root from each field replicate, were used for ginsenoside content quantification. The roots were dried in an oven at 35°C to consistent weight (Li et al., 2019), and the ginsenosides were extracted and quantified as previously described by Li et al (2019; 2021). Briefly, ginsenosides were extracted from the 4-year-old plant roots of each cultivar or landrace, with two replicates. One gram of each dried root replicate sample was used for ginsenoside extraction. The standards used for these ginsenosides were purchased from the National Institute for the Control of Pharmaceutical and Biological Products (Beijing, China). Individual ginsenosides, including Rg1, Re, R0, Rf, Rb1, Rg2, Rh1, Rc, Rb2, Rb3, Rd, F1, Rg3, F2, Rh2, and PPD, were separated and detected using the Waters Alliance HPLC (high-performance liquid chromatography), with e2695 Separation Module. The contents of the 16 ginsenosides were determined using the Waters 2489 Ultraviolet Spectrophotometric Detector (Waters, Milford, MA, USA).

### Correlation analysis between ginsenosides by content in the mini-core collection and their relationships in biosynthesis of the ginsenosides

We performed a correlation analysis between the 16 ginsenosides using their contents in the 4-year-old plant roots of the entire mini-core collection, each subpopulation, and the cultivars and landraces collected from each geographical region. The correlation analyses between ginsenosides were based on the content variations of the ginsenosides in the cultivars and landraces of the entire mini-core collection, each subpopulation, or those collected from each geographical region. The results were then used to deduce their relationships in ginsenoside biosynthesis. The relationships of the ginsenosides by content were determined by Person’s correlation coefficient calculated using the SPSS package (IBM SPSS Statistics 23) and by network analysis based on ginsenoside contents using the BioLayout Express3D (Version 3.3) (Theocharidis et al., 2009). Their potential relationships in ginsenoside biosynthesis were inferred using the correlation coefficients of the ginsenosides in content, along with the network analysis results. We hypothesized that if the content correlation of a pair of ginsenosides is the strongest among all 16 ginsenosides, they are the most likely to interact directly in the ginsenoside biosynthesis pathway. If their correlation in ginsenoside content is weaker, they are likely to interact indirectly in the ginsenoside biosynthesis pathway.

### Comparative analysis of ginsenoside contents between subpopulations and between cultivars or landraces collected from different geographical regions

The ginsenoside contents of the cultivars and landraces clustered into different subpopulations and admixture group or collected from different geographical regions were compared by ANOVA (analysis of variance) with a single factor, followed by Tukey’s HSD (honestly significant difference) test using Excel. A two-tailed significance level was used for the analysis.

### Quantification of gene transcript expressions

As different transcripts spliced from a gene may have different biological functions (Zhang et al., 2019, Zhang et al., 2020b), the expressions of individual gene transcripts in the 4-year-old plant roots of the mini-core cultivars and landraces were quantified with their clean reads by the RSEM (RNA-seq by Expectation Maximization) software (Li and Dewey, 2011) using the 248,993 transcripts previously assembled from 14 tissues of a 4-year-old Jilin ginseng plant (Wang et al., 2015) as the reference. The expressions of the gene transcripts were presented by transcripts per million (TPM) for comparative analysis among the cultivars and landraces.

### Heatmap construction and co-expression network analysis of ginsenoside biosynthesis genes between subpopulations and between cultivars or landraces collected from different geographical regions

Moreover, we examined the expressions and interactions of the 11 genes previously cloned that encode the key enzymes involved in ginseng ginsenoside biosynthesis to further characterize the mini-core collection (for detail of the genes, see Introduction). The expressions of 15 transcripts of the 11 ginsenoside biosynthesis genes were extracted from the expression dataset of the mini-core collection. Expression heatmaps of the 15 gene transcripts were constructed and visualized for the cultivars and landraces collected from different geographical regions and for different subpopulations and the admixture group using an R language package (Makowski et al., 2020). The co-expression networks of the genes were constructed using the BioLayout Express3D Version 3.3 software (Theocharidis et al., 2009). Because the number of cultivars and landraces collected from each geographical region or contained in each subpopulation was different, varying from 42 to 207 for different geographical regions and 35 to 131 for different subpopulations, 30 cultivars and landraces were randomly selected from each geographical region or subpopulation and used for the heatmap and co-expression network analyses. This allowed the resultant heatmaps and networks to be comparable between geographical regions or subpopulations.

### Estimation of relationships between cultivars and landraces

Pearson’s correlation coefficients were calculated pair-wisely between cultivars and landraces of the mini-core collection to estimate their relationships using an R language package (Makowski et al., 2020). The correlation analyses between cultivars and landraces were based on the variations of 10,000 random gene transcript expressions with 10 bootstrap replications, 15 ginsenoside biosynthesis gene transcript expressions, 16 ginsenoside contents, and the representative selection of high-quality genic SNPs. The results were visualized using an R language package (Makowski et al., 2020). The pair-wise relationships of the cultivars and landraces were compared between subpopulations or geographical regions by ANOVA, followed by least significance difference (LSD).

## Results

### Composition of the mini-core collection

A mini-core collection was constructed for Jilin ginseng from 344 cultivars and landraces (**Figure 1**; **Table S1**). Of the 344 cultivars and landraces, 207 (60.2%) were collected from seven major ginseng-producing counties, Baishan; 42 (12.2%) from two major ginseng-producing counties, Jilin; 42 (12.2%) from three major ginseng-producing counties, Tonghua; and 53 (15.4%) from four major ginseng-producing counties, Yanbian (**Figure 1**; **Table S1**). Therefore, the cultivars and landraces of the mini-core collection properly represent the geographical origin and diversity of Jilin ginseng.

### Molecular characteristics of the mini-core collection determined by genic SNPs

**Figure 2:**
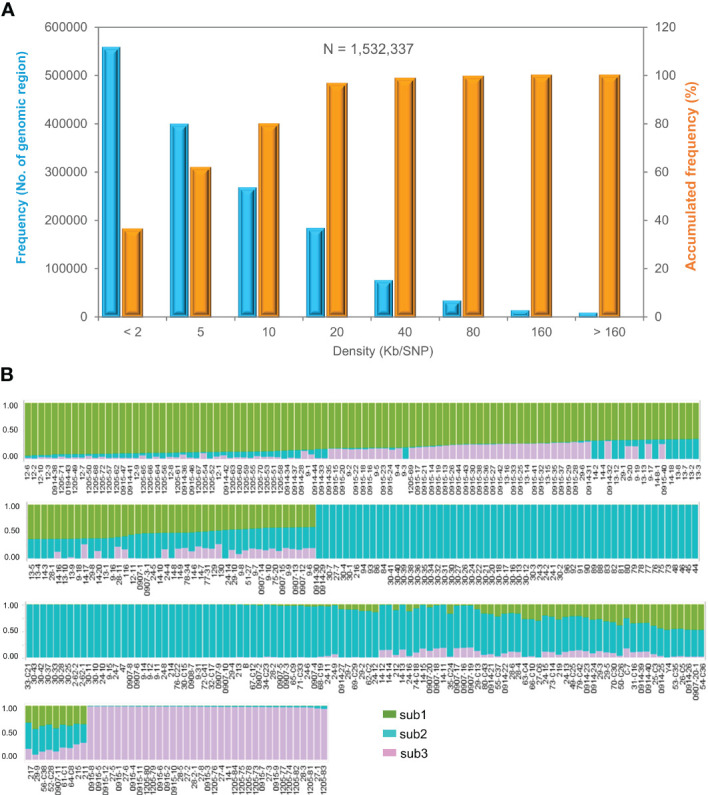
Molecular variation and population structure of the Jilin ginseng mini-core collection. **(A)** Distribution of 1,532,337 SNPs along the ginseng genome. **(B)** The population structure of the mini-core collection at k = 3. Sub1, sub2, and sub3 indicate subpopulations 1, 2, and 3, respectively (for detail, see **Table S1**).

A total of 16.3 billion 150-nucleotide clean reads or over 244 Gb transcriptome sequences were obtained for the transcriptomes of the 344 cultivars and landraces of the mini-core collection, with a range from 40.1 million to 51.5 million clean reads and an average of 47.2 million clean reads per cultivar or landrace. From the transcriptome sequences, 1,532,337 genic SNPs were identified, with an average of 25.6 SNPs per gene, suggesting a high variation of ginseng germplasm. These SNPs spanned 2,761.8 Mb (92.5%) of the 2,984.9 Mb ginseng genome assembly (Kim et al., 2018), with a range from one SNP per less than 2 kb to one SNP per 160 kb and an average of one SNP per 1.8 kb (**Figure 2A**). Of the 1,532,337 SNPs, over 557,000 (36.4%) distributed with a distance of less than 2 kb and over 1.4 million (>97.0%) distributed with a distance of less than 20 kb. We obtained 26,600 high-quality genic SNPs after filtering out those that had a missing data frequency > 10%, MAF < 10%, and base calling quality < Q30. The high-quality genic SNPs also had a distribution similar to that of all 1,532,337 SNPs along the ginseng genome.

### Population structure

The population structure of the mini-core collection was evaluated based on 6,232 SNPs that were chosen from the 26,600 high-quality SNPs, with one SNP per approximately 500 kb along the ginseng genome. The structure of the mini-core collection was estimated using the LnP(K) and ΔK values, respectively, at a number (K) of subpopulations varying from 2 to 10 (**Figure S1**; **Table S1**). When K = 2, the mini-core collection was clustered into two subpopulations and an admixture group, with one subpopulation consisting of 130 of the 344 cultivars and landraces, the other subpopulation consisting of 137 cultivars and landraces, and the admixture group consisting of 77 cultivars and landraces. At K = 3, the mini-core collection was clearly clustered into three subpopulations, defined sub1, sub2, and sub3, and an admixture group. At K = 4, the mini-core collection was clustered into four subpopulations and an admixture group, but the fourth subpopulation consisted of only one of the 344 cultivars and landraces. Therefore, the mini-core collection was finally clustered into three subpopulations and an admixture group (**Figure 2B**). Sub1, sub2, and sub3 of the mini-core collection consisted of 80 cultivars and landraces, with an ancestry index of 0.7010–0.9400; 131 cultivars and landraces, with an ancestry index of 0.7090–0.9970; and 35 cultivars and landraces, with an ancestry index of 0.9430–0.9890, respectively. The admixture group of the mini-core collection contained 98 cultivars and landraces, with an ancestry index of less than 0.7000, of which 66 originated from three ancestries and 32 from two ancestries (**Table S1**). Nevertheless, the subpopulation clustering of the cultivars and landraces was not consistent with their geographical origin, suggesting that extensive migration of the cultivars and landraces has occurred among the geographical regions.

### Phylogeny and principal component analysis

**Figure 3:**
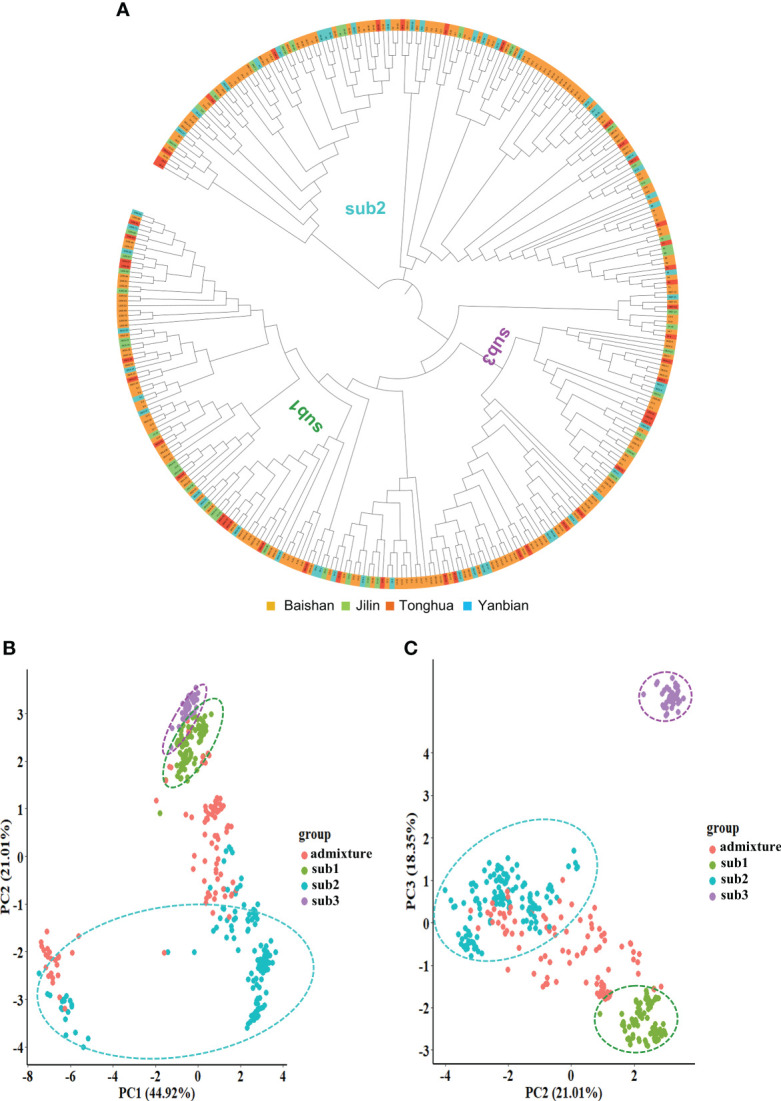
The molecular cladogram **(A)** and principal component (PC) analysis **(B, C)** of the Jilin ginseng mini-core collection. Different colors of the cladogram indicate the geographical regions where the cultivars and landraces were collected. Sub1, sub2, and sub3 indicate subpopulations 1, 2, and 3, respectively (see **Figure 2B**).

Furthermore, we evaluated the molecular relationships among the 344 cultivars and landraces of the mini-core collection by phylogenic analysis using all 26,600 high-quality SNPs (**Figure 3A**). As expected, the mini-core collection was clustered into three main clusters that corresponded to its three subpopulations determined by the STRUCTURE software. No consistency was observed between clustering and geographical collection of the cultivars and landraces. This result was consistent with the result of the population structure analysis for the mini-core collection.

Principal component analysis (PCA) based on all 26,600 high-quality SNPs also verified the population structure of the mini-core collection determined with the 6,232 SNPs (**Figures 3B, C**). The first three principal components (PCs), PC1, PC2, and PC3, explained 84.3% of the mini-core collection genetic variation. The three subpopulations of the mini-core collection, sub1, sub2, and sub3, and admixture group were properly clustered according to the top three PCs. Sub1 and sub3 were separated by PC2 with PC3. Sub2 was largely separated from the admixture group by either PC1 with PC2 or PC2 with PC3. Therefore, the result supported the population structure of the mini-core collection determined based on 6,232 SNPs above.

### Molecular variation and diversity of the mini-core collection

**Figure 4:**
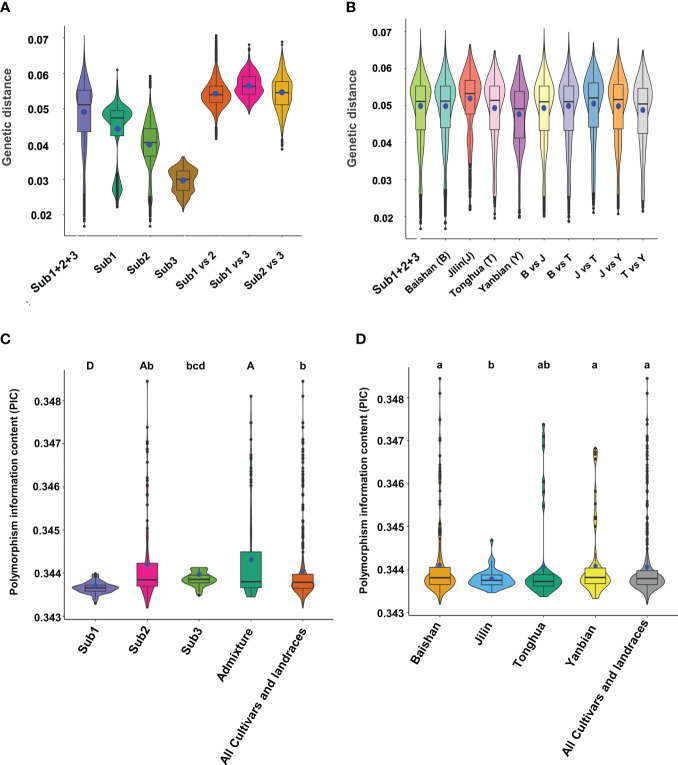
Pairwise genetic distance and polymorphism information content (PIC) of the Jilin ginseng mini-core collection. **(A)** Genetic distances between cultivars or landraces within and between subpopulations (Subs). **(B)** Genetic distances between cultivars or landraces within and between geographical regions where they were collected. Note that the admixture group was excluded from the analysis because they were derived from introgression between subpopulations. Sub1 + 2+3 indicated all cultivars and landraces grouped into subpopulations 1, 2, and 3. **(C)** PICs in the cultivars and landraces clustered into different subpopulations (Subs) and admixture. **(D)** PICs in the cultivars and landraces collected from different geographical regions. The whisker box edges indicate the upper and lower quantiles with the average of the genetic distances or PICs shown by a blue dot and the median value of the genetic distances or PICs shown by a line in the middle of the box. The difference of PIC is indicated by letters, with different letters indicating the difference of PIC at a significance level of *P* ≤ 0.05 for lowercase letters and *P* ≤ 0.01 for capital letters and the same letter indicating no difference of PIC at a significance level of *P* ≤ 0.05.

We deciphered the molecular variation and diversity of the mini-core collection with pairwise genetic distance, allele frequency, gene diversity, heterozygosity, and polymorphism information content (PIC) using 6,232 of the high-quality SNPs selected with one SNP in approximately 500 kb of the ginseng genome. Pairwise genetic distance analysis excluding the admixture group showed that the mini-core collection had a pairwise genetic distance varying from 0.0167 to 0.0707, with a variation of 4.4-fold and an average genetic distance of 0.0488. The pairwise mean genetic distance of the mini-core collection was larger than the within-sub1, within-sub2, and within-sub3 mean genetic distances of 0.0436, 0.0398, and 0.0296, respectively. It was apparent that the pairwise genetic distances between subpopulations were much larger than the within-subpopulation genetic distances (**Figure 4A**). We also analyzed genetic distances of the cultivars and landraces collected from each region and different regions, Baishan (B), Jilin (J), Tonghua (T), and Yanbian (Y). Similar genetic distances were observed for the cultivars and landraces collected from each region and different regions (**Figure 4B**).

The mini-core collection had a mean minor allele frequency of 0.42, with a range from 0.05 to 0.50 and a frequency of <0.10 for 6.0% of the 6,232 SNPs. The gene diversity and heterozygosity of the mini-core collection varied from 0.01 to 0.50 with an average of 0.45 and 0.10 to 1.00 with an average of 0.85, respectively. The PIC varied from 0.1 to 0.38 with an average of 0.34 (**Table S2**). In comparison, the admixture group had the highest PIC that was significantly higher than those of the entire mini-core collection, sub1, and sub3, whereas it was not significantly different from that of sub2 (**Figure 4C**). The cultivars and landraces collected from Baishan and Yanbian had the highest PICs that were significantly higher than those from Jilin but not significantly different from those of the entire mini-core collection and Tonghua (**Figure 4D**).

### Content variations of ginsenosides in the mini-core collection

**Figure 5:**
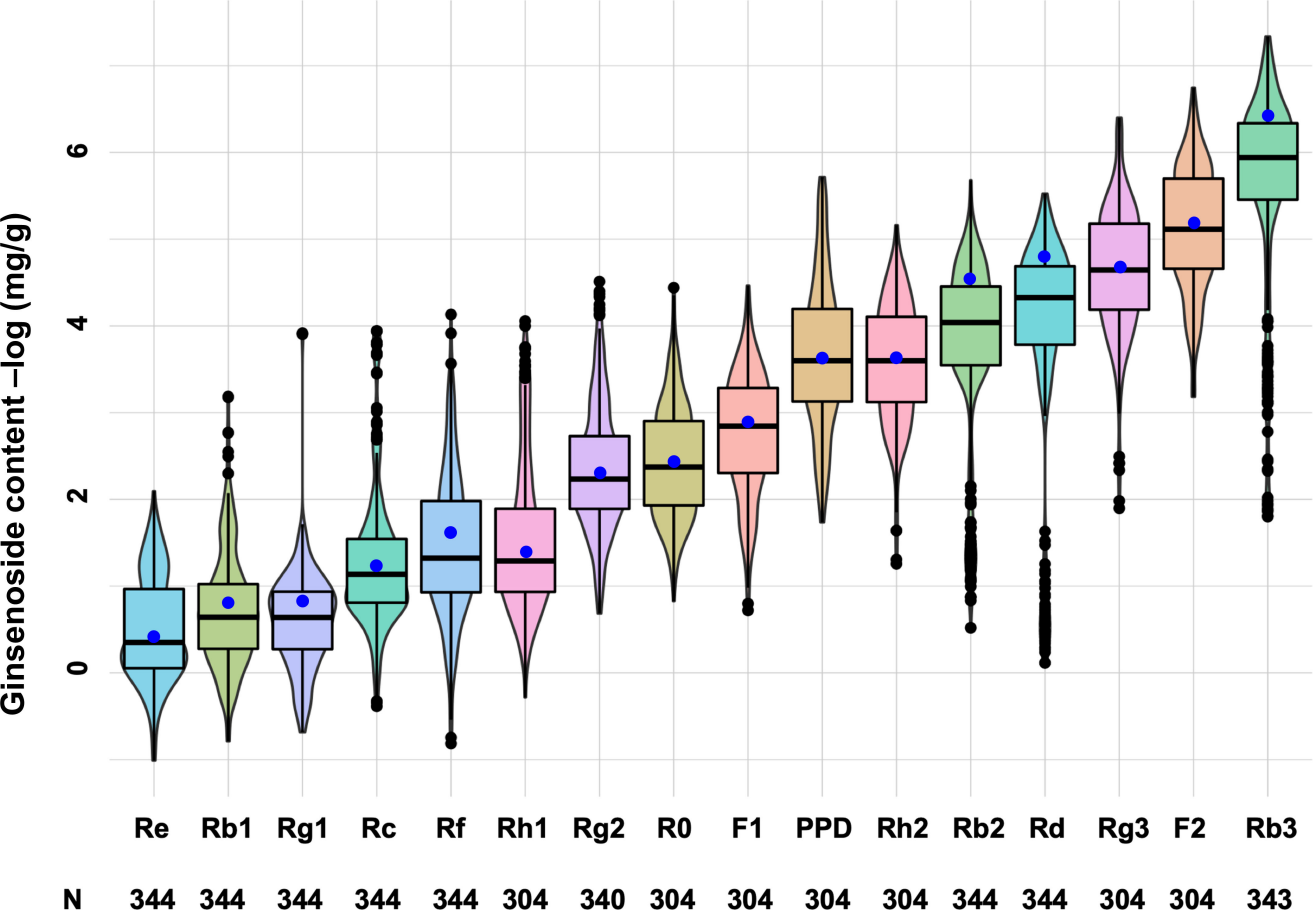
Genetic variation of 16 ginsenoside contents among cultivars and landraces of the mini-core collection. The whisker box edges indicate the upper and lower quantiles with the average of the ginsenoside contents shown by a blue dot and the median value of the ginsenoside contents shown by a line in the middle of the box. N: numbers of the cultivars and landraces analyzed.

The mini-core collection was then characterized with 16 ginsenosides, including Rg1, Re, R0, Rf, Rb1, Rg2, Rh1, Rc, Rb2, Rb3, Rd, F1, Rg3, F2, Rh2, and PPD (**Figure 5**; **Table S3**). The mean contents of the ginsenosides varied widely, from 0.008 mg/g dry matter for F2 to 0.733 mg/g for Re in the 4-year-old plant roots of the mini-core collection. The content of each ginsenoside had a variation range from 0.001 to 0.042 for F2 to from 0.016 to 2.258 for Rf, with a CV (coefficient of variance) from 55.8% for Rg1 to 250.7% for Rb3 among the cultivars and landraces of the collection. Similar variations of the ginsenoside contents were observed among the cultivars and landraces collected from each geographical region (**Table S3**). These results confirmed the genetic diversity and representativeness of the mini-core collection in ginsenoside contents for Jilin ginseng. Finally, we compared the contents of the 16 ginsenosides among the cultivars and landraces collected from Baishan, Jilin, Tonghua, and Yanbian. No significant difference of ginsenoside contents was observed between the cultivars and landraces from different regions (**Figure S2**). This result indicated that extensive migration of Jilin ginseng has occurred among geographical regions.

**Figure 6:**
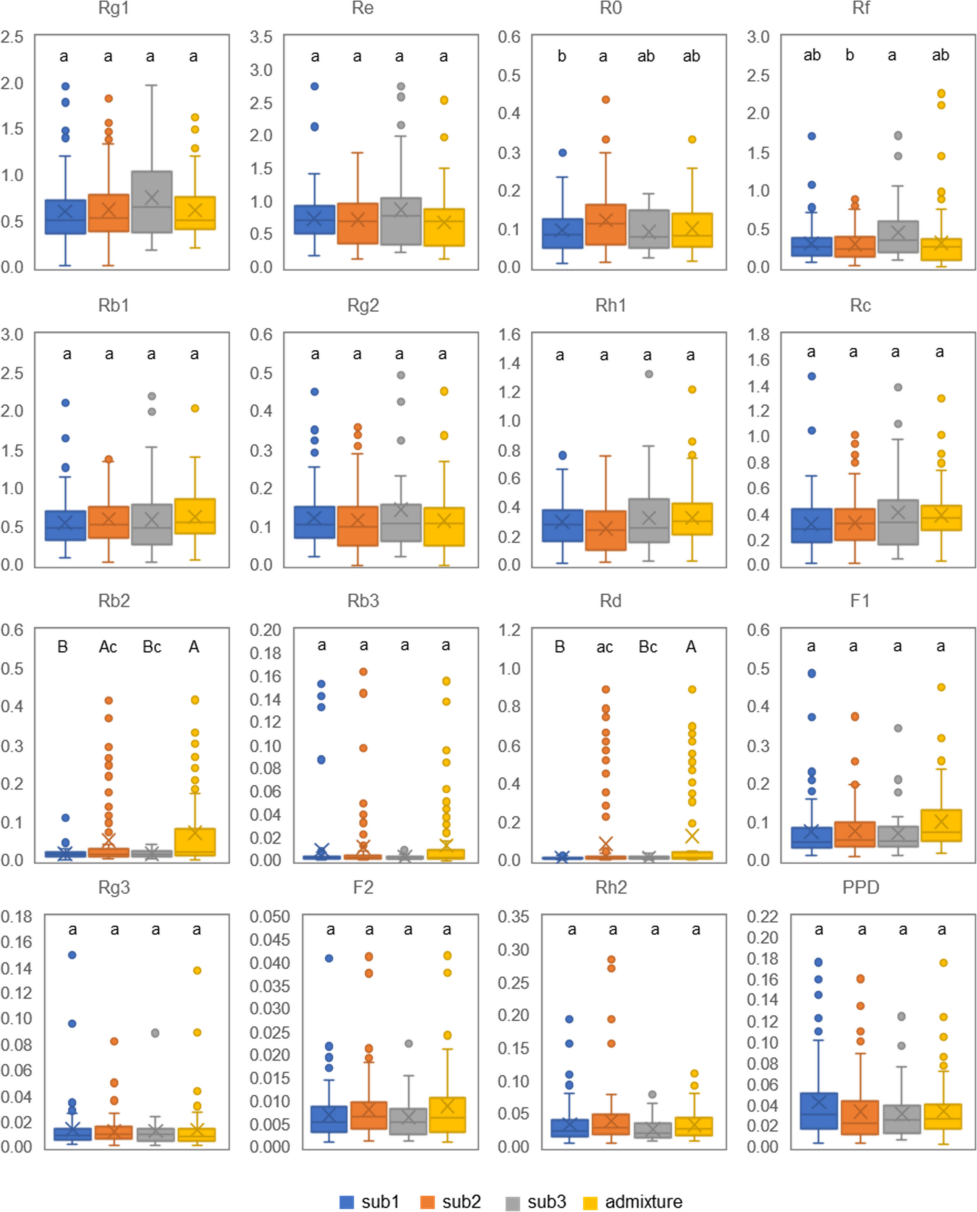
Genetic variation of 16 ginsenoside contents in 4-year-old plant roots among the three subpopulations and admixture group of the Jilin ginseng mini-core collection. The same letters indicate that ginsenoside contents have no significant difference at *P* ≤ 0.05, and different small and capital letters indicate that the differences of ginsenoside contents are significant at *P* ≤ 0.05 and 0.01, respectively, among the subpopulations and admixture (ANOVA and Tukey HSD).

We also compared the content variations of 16 ginsenosides among the three subpopulations and admixture group of the mini-core collection (**Figure 6**; **Table S4**). No significantly different content was observed among the means of sub1, sub2, sub3, and admixture group for Rg1, Re, Rb1, Rg2, Rh1, Rc, Rb3, F1, Rg3, F2, Rh2, and PPD (**Figure 6**) (ANOVA, *P* > 0.05), but their within-subpopulation or admixture group variations were dramatic among cultivars and landraces (**Table S4**). For instance, Rg1 had a within-sub1 content variation ranging from 0.020 to 1.980 mg/g, varying by 99-fold, and with a coefficient of variance (CV) of 61.25%. Rb3 had a content variation for sub1, ranging from 0.001 to 0.153 mg/g, varying by 153-fold, and with a CV of 306.72%. The contents of the four remaining ginsenosides, R0, Rf, Rb2, and Rd, were different among sub1, sub2, sub3, and admixture group (ANOVA, *P* < 0.05). R0 had the highest content in sub2, followed by admixture group, sub1, and sub3. Rf was the most abundant in sub3, and then admixture group, sub1, and sub2 in descending order. The content of Rb2 was the highest in the admixture group, followed by sub2, sub1, and sub3. Rd was also the highest in content in the admixture group, but followed by sub2, sub3, and sub1 (**Figure 6**; **Table S4**).

### Correlations of ginsenosides and their relationships in the biosynthesis of ginsenosides

**Figure 7:**
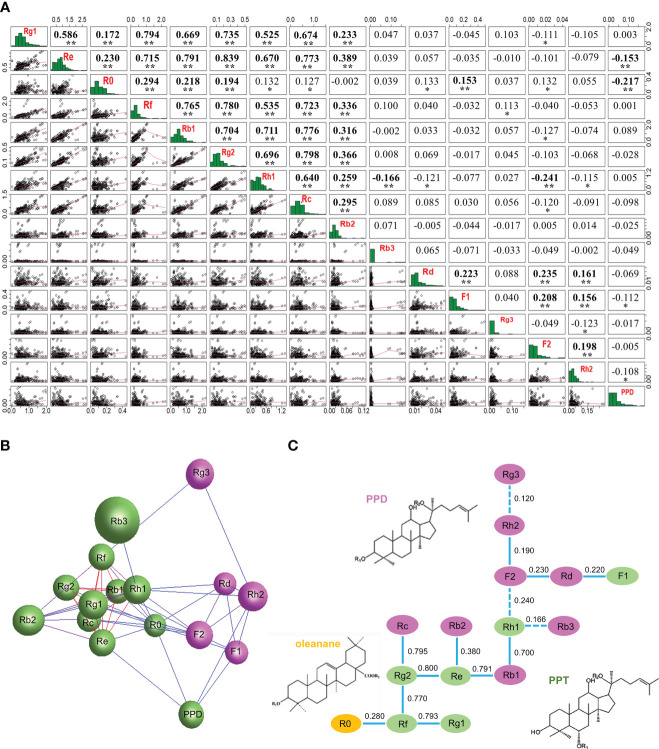
Relationships of 16 ginsenosides inferred by their content variation in the 4-year-old roots of 344 cultivars and landraces. **(A)** Correlations of the ginsenosides in content. “*” and “**” indicate that the correlations are significant at *P* ≤ 0.05 and 0.01, respectively. **(B)** The network of the ginsenosides constructed at *P* ≤ 0.05 based on their contents in the 344 cultivars and landraces. Different colors indicate the different clusters of the ginsenosides. **(C)** The potential relationships of the ginsenosides in the ginsenoside biosynthesis deduced based on their content correlation coefficients, with pink, green, and orange indicating the PPD-typed ginsenosides, PPT-typed ginsenosides, and oleanane-typed ginsenoside, respectively. Solid lines between ginsenosides indicate positive correlations and dot lines indicate negative correlations.

For the entire mini-core collection, correlation analysis showed that 57 (48%) of the 120 possible pairwise correlations of the 16 ginsenosides were significant, 44 of which were significant at *P* < 0.01 and 13 at *P* = 0.05–0.01. Of the 57 correlated ginsenoside pairs, 46 were positively correlated whereas 11 were negatively correlated (**Figure 7A**). Most of the correlative pairs of the ginsenosides were also observed among the cultivars and landraces collected from each geographical region (**Table S5**), thus confirming the correlations of the ginsenosides in the entire mini-core collection. There were 38 (32%) of all 120 possible pairs among the 16 ginsenosides correlated for sub1, 82 (68%) for sub2, 29 (24%) for sub3, and 88 (73%) for the admixture group. The numbers of correlation pairs for sub1 and sub3 were smaller, and those of sub2 and the mixture group were larger than the 57 (48%) pairs of correlations for the entire mini-core collection (**Table S6**; **Figure 7A**). Interestingly, the content of F2 was independent of the contents of all 15 other ginsenosides and the content of R0 was correlated with that of only F1 in sub3. In contrast, the content of F2 was correlated with those of seven, six, and eight of the 15 other ginsenosides and the content of R0 was correlated with those of two, 13, and 14 of the 15 other ginsenosides in sub1, sub2, and the admixture group. The numbers of ginsenosides with which F2 was correlated were larger than the eight ginsenosides for the entire mini-core collection, but the numbers of ginsenosides with which R0 was correlated were smaller than or like those of ginsenosides for the entire mini-core collection.

When their contents were used for network analysis, the 16 ginsenosides formed a single network consisting of two clusters (**Figure 7B**) at *P* ≤ 0.05, with each ginsenoside strongly correlated with one or more other ginsenosides. This result suggested that they were synthesized through a single complicated pathway or multiple pathways that are correlated in ginsenoside biosynthesis (Zhang et al., 2020b; Jiang et al., 2022). One of the two clusters was made of four PPD-typed ginsenosides and one PPT-typed ginsenoside, and the other contained five PPT-typed ginsenosides, five PPD-typed ginsenosides, and one oleanane-typed ginsenoside.

The relationship analysis of the 16 ginsenosides in the biosynthesis of ginsenosides revealed that the PPT-typed ginsenoside, Rg2, was strongly correlated with PPT-typed ginsenosides, Re and Rf; and Rf with PPT-typed ginsenoside, Rg1; with a correlation coefficient of 0.800, 0.770, and 0.793, respectively. The PPT-typed ginsenoside, Rg2, was strongly correlated with the PPD-typed ginsenoside, Rc; the PPT-typed ginsenoside, Re, with the PPD-typed ginsenoside, Rb1; and the PPT-typed ginsenoside, Rh1, with the PPD-typed ginsenoside, Rb1; with correlation coefficients of 0.795, 0.791, and 0.700, respectively. These results suggested that these seven ginsenosides were highly likely in a closer position in the ginsenoside biosynthesis pathway. Correlations were also observed among the remaining eight ginsenosides (*P* < 0.05), including six PPD-typed ginsenosides, one PPT-typed ginsenoside, and one oleanane-typed ginsenoside, but with a lower correlation coefficient varying from 0.120 to 0.380, suggesting that these eight ginsenosides are likely in a more distant position or existence of one or more other ginsenosides between them in the ginsenoside biosynthesis pathway (**Figure 7C**).

### Expressions and networks of genes encoding key enzymes involved in ginsenoside biosynthesis

**Figure 8:**
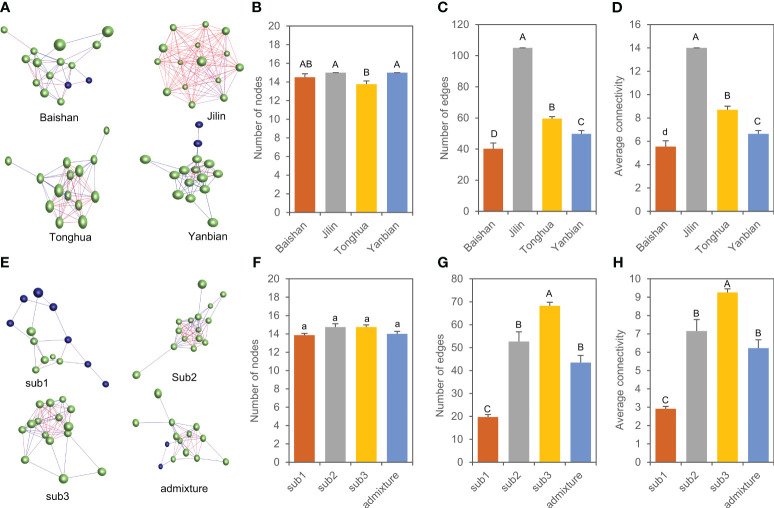
Co-expression network variation of 15 gene transcripts involved in biosynthesis of ginsenosides in 4-year-old plant roots. **(A–D)** Networks of the genes **(A)** and their variation in number of nodes **(B)**, number of edges **(C)**, and connectivity **(D)** among cultivars and landraces collected from different geographical regions. **(E–H)** Networks of the genes **(E)** and their variation in number of nodes **(F)**, number of edges **(G)**, and connectivity **(H)** among the three subpopulations and admixture group. The same letters indicate that the difference is not significant at *P* ≤ 0.05, and different lowercase and capital letters indicate that the differences are significant at *P* ≤ 0.05 and 0.01 (ANOVA and Tukey HSD), respectively. Each ball represents a gene node; the line between genes is the interaction edge; different color indicates different clusters of the network.

A total of 11 genes that were spliced into 15 transcripts encoding key enzymes controlling ginsenoside biosynthesis have been reported in ginseng (see Introduction). To have a glimpse into variation of the expression activities and interactions of these genes in the mini-core collection, we constructed their expression heatmaps and co-expression networks in 4-year-old plant roots of different subpopulations and admixture group and cultivars and landraces collected from different geographical regions. Comparative analysis of the heatmaps showed that the genes expressed in low activity in almost all cultivars and landraces analyzed, regardless of their geographical regions and there was no tendency that the cultivars and landraces collected from a similar geographical region were grouped into a similar cluster (**Figure S3**). Nevertheless, the cultivars and landraces of each subpopulation somehow tended to be grouped into a cluster (**Figure S4**). Co-expression networks showed that the genes tended to form a co-expression network in the cultivars and landraces of both each geographical region (**Figure 8A**) and each subpopulation or admixture group (**Figure 8E**). The numbers of the gene nodes, numbers of interaction edges, and connectivity or robustness of the network were different among the cultivars and landraces collected from different geographical regions (*P* < 0.01), thus distinguishing the origin of the cultivars and landraces among geographical regions. Among the four geographical regions, the cultivars and landraces from Jilin had the network with the largest number of gene nodes, the largest number of gene interaction edges, and the highest connectivity (**Figures 8B–D**). Among the subpopulations and admixture group of the mini-core collection, there was no difference in number of gene nodes, but the number of gene interaction edges and connectivity were different (*P* < 0.01), differing the cultivars and landraces among subpopulations and admixture group. Subpopulation 3 (sub3) had the largest number of gene interaction edges and the highest connectivity (**Figures 8F–H**).

### Relationships between cultivars and landraces of the mini-core collection

**Figure 9:**
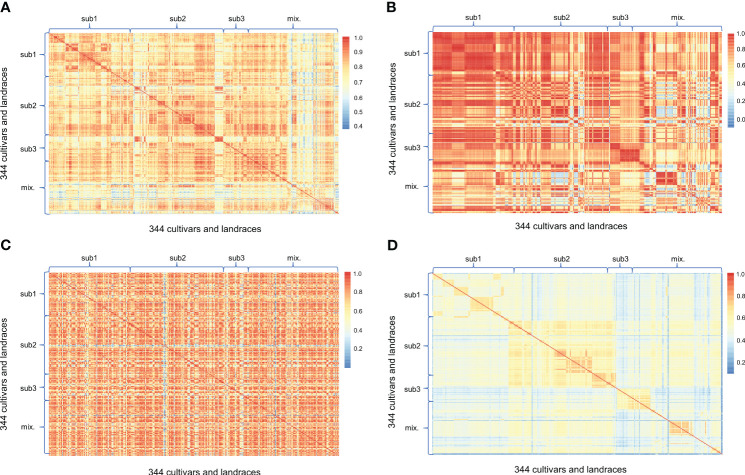
Relationships between the cultivars and landraces clustered into different subpopulations determined using different datasets. **(A)** Relationships between the cultivars and landraces determined by the expressions of 10,000 transcripts randomly selected from the transcripts expressed in 4-year-old plant roots, with 10 bootstrap replications. The means of the relationships were used to construct the figure. **(B)** Relationships between the cultivars and landraces determined by the expressions of 15 genes encoding key enzymes involved in ginsenoside biosynthesis in 4-year-old plant roots. **(C)** Relationships between the cultivars and landraces determined by the contents of 16 mono-ginsenosides in 4-year-old plant roots. **(D)** Relationships between the cultivars and landraces determined by 6,232 high-quality genic SNPs. The Pearson’s correlation coefficients between the cultivars and landraces were used to present their relationships. The correlation analyses were based on variation of different gene transcript expressions, different ginsenoside contents, or different SNP genotypes in each cultivar or landrace. “Sub” for subpopulation; “mix.” for admixture.

Finally, we examined the relationships between cultivars and landraces of the mini-core collection by subpopulation and by geographical region using the expressions of 10,000 random gene transcripts with 10 bootstrap replications, the expressions of 15 gene transcripts that encode key enzymes involved in ginsenoside biosynthesis, the contents of 16 ginsenosides, and the genotypes of 6,232 high-quality genic SNPs, respectively (**Figure 9**; **Figures S5** and **S6**). By subpopulation, when the expressions of 10,000 random gene transcripts were used to determine the relationships between the cultivars and landraces, minor kinships were observed between some of the cultivars and landraces that had high correlations (**Figure 9A**). The correlations between cultivars and landraces varied from 0.40 to 0.99 (when *P* = 0.05, *r* = 0.0196), with an average of 0.78 and a CV of 12.66% (**Figure S6A**). The relationships between cultivars and landraces among subpopulations and admixture were subpopulation 3 > subpopulation 1 > subpopulation 2 > the entire mini-core collection > admixture (*P* < 0.01) (**Figure S6A**). When the expressions of 15 gene transcripts that encode key enzymes involved in ginsenoside biosynthesis were used to determine the relationships between the cultivars and landraces, some distinguished kinships were identified (**Figure 9B**). The variation of their correlations ranged from 0.00 to 1.00 (when *P* = 0.05, *r* = 0.5140), with an average of 0.76 and a CV of 31.29% (**Figure S6B**). The relationships between cultivars and landraces were subpopulation 1 > subpopulation 3 > subpopulation 2 > the entire mini-core collection > admixture (*P* < 0.01) (**Figure S6B**). When the contents of 16 ginsenosides were used to determine the relationships between the cultivars and landraces, no clear kinship was identified (**Figure 9C**). The variation of correlations between them ranged from 0.01 to 1.00 (when *P* = 0.05, *r* = 0.4973), with an average of 0.73 and a CV of 25.66% (**Figure S6C**). The relationships between cultivars and landraces were subpopulation 2 > the entire mini-core collection, admixture > subpopulation 1 > subpopulation 3 (*P* < 0.01) (**Figure S6C**). When the genotypes of 6,232 high-quality genic SNPs were used to determine the relationships between the cultivars and landraces, some distinguished kinships were identified (**Figure 9D**). The variation of their correlations ranged from 0.12 to 0.83 (when *P* = 0.05, *r* = 0.0248), with an average of 0.56 and a CV of 18.13% (**Figure S6D**). The relationships between cultivars and landraces were subpopulation 3 > subpopulation 2 > the entire mini-core collection > subpopulation 1 > admixture (*P* < 0.01) (**Figure S6D**). Nevertheless, no clear consistency was observed among the relationships revealed by random gene transcript expressions, ginsenoside biosynthesis gene transcript expressions, ginsenosides, and random genic SNPs.

By geographical region, minor kinships were identified between cultivars and landraces using the expressions of 10,000 random gene transcripts (**Figure S5A**) and the genotypes of 6,232 high-quality genic SNPs (**Figure S5D**), respectively, but no kinship was identified using either the expressions of 15 gene transcripts that encode key enzymes involved in ginsenoside biosynthesis (**Figure S5B**) or the contents of 16 ginsenosides (**Figure S5C**). Similar to those compared by subpopulation, no consistent pattern of relationships was obtained by these four characters. The relationships of the cultivars and landraces among geographical regions revealed by the expressions of 10,000 random gene transcripts were Jilin > Baishan and the entire mini-core collection > Tonghua > Yanbian (*P* < 0.01) (**Figure S6E**); those revealed by the expressions of 15 gene transcripts that encode key enzymes involved in ginsenoside biosynthesis were Jilin > Tonghua > Yanbian and the entire mini-core collection > Baishan (*P* < 0.05) (**Figure S6F**); those revealed by the contents of 16 ginsenosides were Yanbian > Baishan and the entire mini-core collection > Tonghua > Jilin (*P* < 0.05) (**Figure S6G**); and those revealed by the genotypes of 6,232 high-quality genic SNPs were Baishan > Tonghua and the entire mini-core collection > Jilin and Yanbian (*P* < 0.05) (**Figure S6H**).

## Discussion

Ginseng is widely used for healthy food and medicine; however, its germplasm research is far behind those of crops, which greatly influences ginseng research and breeding. Mini-core collection or panels of representative germplasm lines have been instrumental for GWAS of agronomic traits and significantly advanced functional genomics research and breeding in crops, such as maize (Flint-Garcia et al., 2005), rice (Yan et al., 2007), cotton (Fang et al., 2017; Ma et al., 2018), and wheat (Khan et al., 2022). Although ginseng germplasm has been investigated (Wang et al., 2015; An et al., 2017; Lee et al., 2020a, Lee et al., 2020b), no mini-core collection that is representative of genetic diversity and variation of ginseng and suitable for GWAS in ginseng has been reported. The present study has, for the first time, constructed such a mini-core collection and characterized it using genic nucleotide sequence variation, ginsenoside content, and gene expression. This mini-core collection has been shown to have a proper representation for ginseng grown in Jilin Province, China, where over 56% of the world’s ginseng are produced (Baeg and So, 2013; Chen et al., 2022). Therefore, the mini-core collection is suitable for genetic and molecular dissection of most, if not all, of traits important to ginseng and useful for genome-wide identification of loci controlling ginsenoside biosynthesis and many other economical traits. The mini-core collection largely consists of cultivars that are currently grown for ginseng production and landraces important for ginseng genetic improvement. The findings and genomic tools developed with the mini-core collection can be immediately and efficiently translated into ginseng genetic improvement and enhanced production. Therefore, the mini-core collection promotes genomics research and facilitates genetic improvement in ginseng.

Functional genes and their nucleotide sequence variations, such as SNPs and InDels, are crucial to functional genomics research and applications of the research results for enhanced plant breeding (Sikora et al., 2011; Liu et al., 2020; Zhang et al., 2020a; Zhang et al., 2021a; Liu et al., 2022a; Liu et al., 2022b; Zhang et al., 2022). This study has generated over 16 billion 150-nucleiotide clean reads or 244 Gb transcriptome sequences for the Jilin ginseng germplasm mini-core collection, from which over 1.5 million genic SNPs have been identified. RNA-seq (Liang and Schnable, 2016; Liu et al., 2020; Zhang et al., 2020a; Jehl et al., 2021; Thorstensen et al., 2021; Liu et al., 2022a; Liu et al., 2022b; Zhang et al., 2022), whole-genome GBS (Fang et al., 2017; Ma et al., 2018), and ddRAD-seq (Song et al., 2021) have been used to generate genome-wide SNPs for genetic studies and breeding. However, unlike RNA-seq that also allows quantification of gene expressions, whole-genome GBS and ddRAD-seq provide no information on gene or transcript expressions that have been widely used for candidate gene identification (Jiang et al., 2022) and phenotype prediction for progeny selection in molecular breeding (Liu et al., 2020; Zhang et al., 2020a; Liu et al., 2022a; Liu et al., 2022b; Zhang et al., 2022). Whole-genome GBS can generate more SNPs well distributed along a genome with a smaller missing data rate, but it is costly. Since ddRAD-seq sequences only the DNA associated with the restriction sites of a restriction enzyme of interest, it is economical, but the numbers of SNPs generated are limited, often fewer than 10,000 quality SNPs, and a large portion of them have missing data for the lines used for genetic studies (Song et al., 2019; Dogan et al., 2023). This number of SNPs is likely insufficient for GWAS as GWAS panels, such as the mini-core collection of ginseng reported herein, have much smaller recombinant segments than bi-parental populations used for genetic mapping. Although the numbers of genic SNPs identified by RNA-seq are fewer and probably less well distributed along a genome, due to an uneven distribution of genes in a genome, than those generated by whole-genome GBS, they are much larger, often approaching tens of thousands to a million quality SNPs (Liu et al., 2020; Zhang et al., 2020a; Liu et al., 2022a; Liu et al., 2022b; Zhang et al., 2022), and the numbers of SNPs with missing data are much fewer than the SNPs generated by ddRAD-seq. Moreover, unlike genome molecular mapping for which genome-wide well-distributed SNPs are desirable, GWAS aims to identify SNPs associated with genes controlling traits of interest. Therefore, the genome-wide even distribution of SNPs does not seem critical to GWAS, because the genome regions having no genic SNPs do not have genes. The genic SNPs and gene expressions resulting from RNA-seq provide not only a deep insight into molecular diversity, population structure, and evolution of the ginseng species as reported in the present study but also genic resources and tools for subsequent ginseng functional genomics research, such as genome-wide identification of genes involved in ginsenoside biosynthesis (Li and Ritchie, 2021; Jiang et al., 2022), and ginseng breeding, such as marker-assisted selection and gene-based breeding (Liu et al., 2020; Zhang et al., 2020a; Zhang et al., 2021a; Liu et al., 2022a).

We have identified 26,600 high-quality SNPs and determined the population structure, phylogeny, and principal components (PCs) of the mini-core collection using the genic SNPs. The mini-core collection has been clustered into three subpopulations and an admixture group using a selection of 6,232 SNPs, which is in consistency with the results of phylogenetic analysis and PCA determined with all 26,600 high-quality SNPs. This indicates that the population structure obtained in this study is highly reliable. Although the population structure of the mini-core collection was weaker with a low ΔK value, relative to other plant species such as rice (Song et al., 2021), similar results were consistently observed for ginseng by other researchers (An et al., 2017; Lee et al., 2020a; Lee et al., 2020b). This observation could be attributed to the limited distribution of ginseng germplasm lines investigated by the present and previous (An et al., 2017; Lee et al., 2020a; Lee et al., 2020b) studies in Changbai Mountains. The optimal number of subpopulations (K = 3) for the mini-core collection was the same as that of the 73 ginseng accessions collected from Jilin Province, China, determined by An et al. (2017), but the subpopulation classification of the mini-core collection is much more accurate than that of An et al. (2017), simply because of the larger number of the high-density SNPs used in the present study. Nevertheless, the optimal number of subpopulations for the mini-core collection is different from those of the ginseng accessions collected from South Korea and Jilin Province, China (Lee et al., 2020a), and South Korea only (Lee et al., 2020b), with K = 12 and K = 7, respectively. The difference could be attributed to the use of a smaller number of DNA markers (17 and 33) for the studies of Lee et al (2020a; 2020b). and/or the different panels of ginseng analyzed.

The cultivars and landraces of the mini-core collection were collected from four geographical regions of the origin and diversity center of Jilin ginseng, but only a limited relationship was observed between the subpopulations and geographical regions from which they were collected. This result was previously observed by An et al. (2017) and Lee et al. (2020b). The inconsistency between subpopulation classification and geographical origin suggests that the cultivars and landrace of the mini-core collection likely originated in three independent geographical regions, from which each subpopulation originated, in Jilin Province, China, followed by extensive migration for ginseng production among these regions.

This study has examined the genetic diversity of the mini-core collection by major allele frequency, heterozygosity, gene diversity, and polymorphism information content (PIC) based on 6,232 genic SNPs. An et al. (2017) studied the genetic diversity of Jilin ginseng using 73 accessions collected from six ginseng production counties with eight SSRs and the same parameters. Of the 73 accessions, 44 (60%) were collected from the same counties as four of the 16 counties collected for the mini-core collection. In comparison, the major allele frequency of the mini-core collection is similar to the mean major allele frequency of 0.57 estimated by An et al. (2017). However, the heterozygosity of the mini-core collection is much higher than the mean heterozygosity of 0.324 revealed by An et al. (2017). Both gene diversity and PIC of the mini-core collection are lower than the results of An et al. (2017). These findings may be attributed to the fact that most, if not all, of ginseng cultivars currently grown in Jilin Province are traditional, not having been subjected to modern breeding. The differences of the present results from those of An et al. (2017) could be due to the differences in population constitution and number and type of markers used.

Ginsenosides are the most important bioactive components in ginseng for healthy food and medicine; therefore, they are widely used to evaluate ginseng quality. However, variation of ginsenoside contents across genotypes, environments, ages, seasons, and tissues (Lee et al., 2017; Kim et al., 2018; Xiu et al., 2019; Jiang et al., 2022) makes it difficult to compare ginsenoside contents among genotypes. This study collected seeds from different cultivars and landraces from different geographical regions and planted them at the same ginseng research experiment stations for ginsenoside content analysis. This experimental design allowed comparison of ginsenoside contents among cultivars and landraces collected from different regions or clustered into different subpopulations, whereas other factors potentially influencing ginsenoside contents of a cultivar, such as environments, ages, seasons, and tissues, were under proper control. The contents of the ginsenosides analyzed in this study have been found to vary dozens-fold among cultivars and landraces of either the entire mini-core collection or individual geographical region. Nevertheless, no significant difference is found in the mean content of every ginsenoside among the four major ginseng-producing geographical regions in Jilin, China. This could be attributed to extensive migration and exchange of cultivars and landraces among the regions. Among the three subpopulations and the admixture group, similar contents have been observed for 12 of the 16 ginsenosides, including Rg1, Re, Rb1, Rg2, Rh1, Rc, Rb3, F1, Rg3, F2, Rh2, and PPD. Only four, namely, R0, Rf, Rb2, and Rd, were significantly different. Nevertheless, every subpopulation or admixture group contains several cultivars or landraces with exceptionally high contents in 10 or more of the 16 ginsenosides studied. These findings suggest that high-quality cultivars of one or more ginsenosides could be directly selected from the existing cultivars or landraces for ginseng production and provide knowledge necessary for efficient ginseng breeding. Ginsenoside contents have been previously studied extensively by other researchers. Xiu et al. (2019) planted cultivated Jilin ginseng seeds to four locations in Jilin Province, China, and analyzed effects of different aged ginsengs and different environments on contents of 14 ginsenosides in roots collected in July and August. Of the 12 ginsenosides that were also measured in our study, the contents of 10, Re, Rg1, Rf, Rb1, Rg2, Rh1, Rc, Rb3, Rd, and Rg3, were consistent and two, R0 and Rb2, were different between our study and Xiu et al. (2019) with same-aged ginseng roots grown in the same region (Baishan), even though we collected samples in September. Kim et al. (2018) studied seasonal content variation of nine ginsenosides in the roots of 5-year-old Korean ginseng grown at a single location. Of the nine ginsenosides also analyzed in our study, the contents of four, Re, Rf, Rg2, and Rd, in our study agreed with those of Kim et al. (2018) sampled in September, but the contents of five, Rg1, Rb1, Rc, Rb2, and R0, measured by our study were lower than those of Kim et al. (2018). The difference could be attributed to the age difference of the samples and/or different environments, in addition to different genotypes, between the two studies.

The present study has, for the first time, revealed that extensive correlations exist in content among the 16 ginsenosides in the mini-core collection, and when they were subjected to network analysis, they have formed only a single network consisting of two clusters and with each being correlated with at least one of the other ginsenosides. These results suggest that the ginsenosides are synthesized through a single or multiple related biological pathways and reflect their relationships in the pathway. However, variation of the correlations has been observed among the cultivars and landraces collected from different geographical regions. Because the samples used for the ginsenoside assay were collected from the cultivars and landraces grown from the same trials, the variation of the ginsenoside content correlations might result from genetic difference of the cultivars and landraces collected from different geographical regions. The correlations of the ginsenosides provide a clue useful for construction of the pathway of ginsenoside biosynthesis and information necessary for breeding varieties of high quality in multiple ginsenosides. Therefore, we have inferred the relationships of the ginsenosides in the pathway of ginsenoside biosynthesis, which is helpful for comprehensively deciphering the molecular mechanisms underlying ginsenoside biosynthesis.

There were 11 genes involved in ginsenoside biosynthesis that have previously been cloned, namely, *β-AS*, *CAS*, *DS*, *FPS*, *SE*, *SS*, *UGT71A27*, *UGT74AE2*, *CYP716A53v2*, *CYP716A52v2*, and *CYP716A47* (Kushiro et al., 1998; Lee et al., 2004; Han et al., 2006; Kim et al., 2009; Han et al., 2011; Kim et al., 2011; Han et al., 2012; Han et al., 2013; Jung et al., 2014; Kim et al., 2014; Han et al., 2020). This study revealed that the expressions of these genes varied in the 4-year-old plant roots across cultivars but had a wide range from low to high levels of expressions. This result is supported by previous studies (Xue et al., 2019; Fang et al., 2022; Wang et al., 2022). Because the genes involved in ginsenoside biosynthesis were several times more likely to form a single co-expression network (Zhang et al., 2020b), this study, for the first time, comparatively analyzed the co-expression networks of the genes to further characterize the cultivars and landraces of ginseng clustered into different subpopulations or collected from different regions. These genes formed a co-expression network in the 4-year-old roots of the cultivars and landraces of either a subpopulation or collected from a geographical region, suggesting their correlation in ginsenoside biosynthesis. However, the co-expression networks of the genes varied dramatically, especially in number of gene interaction edges and connectivity, among not only subpopulations but also the cultivars and landraces collected from different geographical regions. These findings may be used to characterize ginseng with different ancestries or produced in different geographical regions.

Knowledge of the relationships between cultivars, landraces, and germplasm lines is crucial to understanding and use of the germplasm collection for advanced ginseng research and breeding. The present study has, for the first time, examined the relationships between ginseng cultivars and landraces using genome-wide gene transcript expressions, ginsenoside biosynthesis gene transcript expressions, ginsenoside contents, and genic SNPs. Of these characters, the relationships of all pairs of the cultivars and landraces revealed by either expressions of 10,000 random gene transcripts or 6,232 high-quality genic SNPs were significant (*P* < 0.01), even though they had a wide variation among the cultivar and landrace pairs. These results indicate that the cultivars and landraces of Jilin ginseng currently used in production and breeding are substantially related with each other in both gene expression and mutations. Nevertheless, the patterns of their relationships revealed were not consistent between the gene transcript expressions and genic SNPs. Although the inconsistency could be partly attributed to the facts that gene expressions are consequences of the interactions of multiple factors, such as genotype-by-environment, gene-by-non gene elements, nucleotide methylation, chromatin modification, and small RNA regulation, whereas SNPs are relatively stable across these factors, it would be rather more likely to reflect different aspects of relationships between the cultivars and landraces. The relationships of 83%–89% of the cultivar and landrace pairs revealed by expressions of 15 ginsenoside biosynthesis gene transcripts or the contents of 16 ginsenosides were significant (*P* < 0.05), thus confirming the general conclusion that the cultivars and landraces of Jilin ginseng tended to be related. These results indicate that the expressions of the ginsenoside biosynthesis genes as well as the biosynthesis of the ginsenosides in which the genes are involved somehow are related or involved in a single or related processes. Moreover, it should be pointed out that the relationships do not seem to dent the robustness and applications of the mini-core collection for advanced ginseng research as this study did not identify significant kinships among the cultivars and landraces. Finally, the relationships of the cultivars and landraces revealed by any of genome-wide gene transcript expressions, ginsenoside biosynthesis gene transcript expressions, ginsenoside contents, and genic SNPs were significantly different between subpopulations of the mini-core collection, or the cultivars and landraces collected from different geographical regions.

## Conclusion

A mini-core germplasm collection of a species is necessary for its advanced genetics and genomics research, gene discovery, genomic tool development, and efficient genetic improvement. We have developed the first mini-core collection for Jilin ginseng, which consists of 344 cultivars and landraces representing the variation and diversity of Jilin ginseng. Transcriptome sequence analysis of the mini-core collection reveals that the genes of ginseng germplasm dramatically vary at the nucleotide sequence level, containing over 1.5 million SNPs. Of the genic SNPs, over 97% have a distance of less than 20 kb. A total of 26,600 high-quality SNPs were identified from the genic SNPs and used to characterize the mini-core collection. The mini-core collection has been clustered into three subpopulations and an admixture group, with a within-subpopulation genetic distance of 0.0296–0.0436, and a between-subpopulation genetic distance of 0.0542–0.0565. Analysis of 16 ginsenosides shows that the mini-core collection also has a wide variation in the content of every ginsenoside studied and each is correlated with at least one of the other ginsenosides. This result suggests that the ginsenosides are synthesized through a single or multiple functionally related pathways. Although the mean contents of most of the 16 ginsenosides are similar among the subpopulations and admixture group, each subpopulation or admixture group has a wide and unique variation for every ginsenoside. Profiling the expressions of the genes cloned to date that encode key enzymes involved in ginsenoside biosynthesis reveals that they generally have a low level of expressions in the cultivars and landraces. The transcripts of the genes formed a co-expression network in the cultivars and landraces of either a subpopulation or from a geographical region, but their network structures varied dramatically among them. When the mini-core collection was pair-wisely deciphered with different genetic or molecular factors, predominant correlations were identified and significant differences of relationships were detected between the subpopulations and admixture, and between the cultivars and landraces collected from different geographical regions. These findings provide information and genetic resources useful for genome-wide identification of genes involved in ginsenoside biosynthesis and development of genomic tools for enhanced research and genetic improvement in ginseng.

## Data availability statement

The datasets presented in this study can be found in online repositories. The names of the repository/repositories and accession number(s) can be found below: https://www.ncbi.nlm.nih.gov/bioproject/PRJNA302556.

## Author contributions

MPZ and YW conceived and designed the project, and MPZ revised and edited the manuscript. SL and YFW wrote the manuscript draft. HH, PC, JL, and YJ extracted and quantified ginsenosides. SL, LL, KW, and MZZ extracted, purified, and quantified the RNA. SL, MC, YH, and LZ conducted data analysis. All authors contributed to the article and approved the submitted version.
