# Supplementary figures and images for: Genetic and molecular dissection of ginseng (Panax ginseng Mey.) germplasm using high-density genic SNP markers, secondary metabolites, and gene expressions
Source: Front Plant Sci. 2023 Jul 28;14:1165349. doi: 10.3389/fpls.2023.1165349 (PMC10416250; doi:10.3389/fpls.2023.1165349)

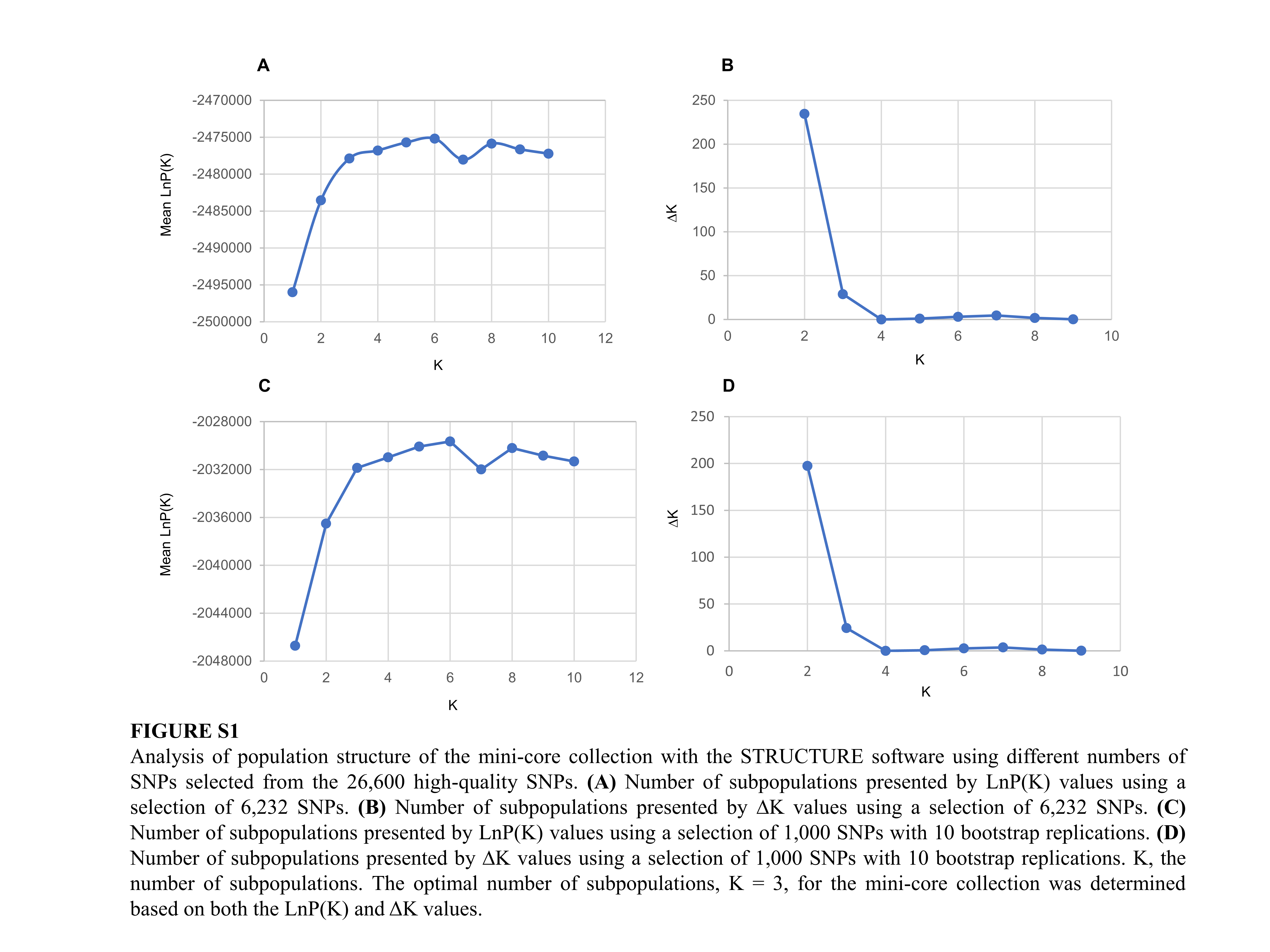

Supplement: Supplementary file 1 [file Image_1.tif]

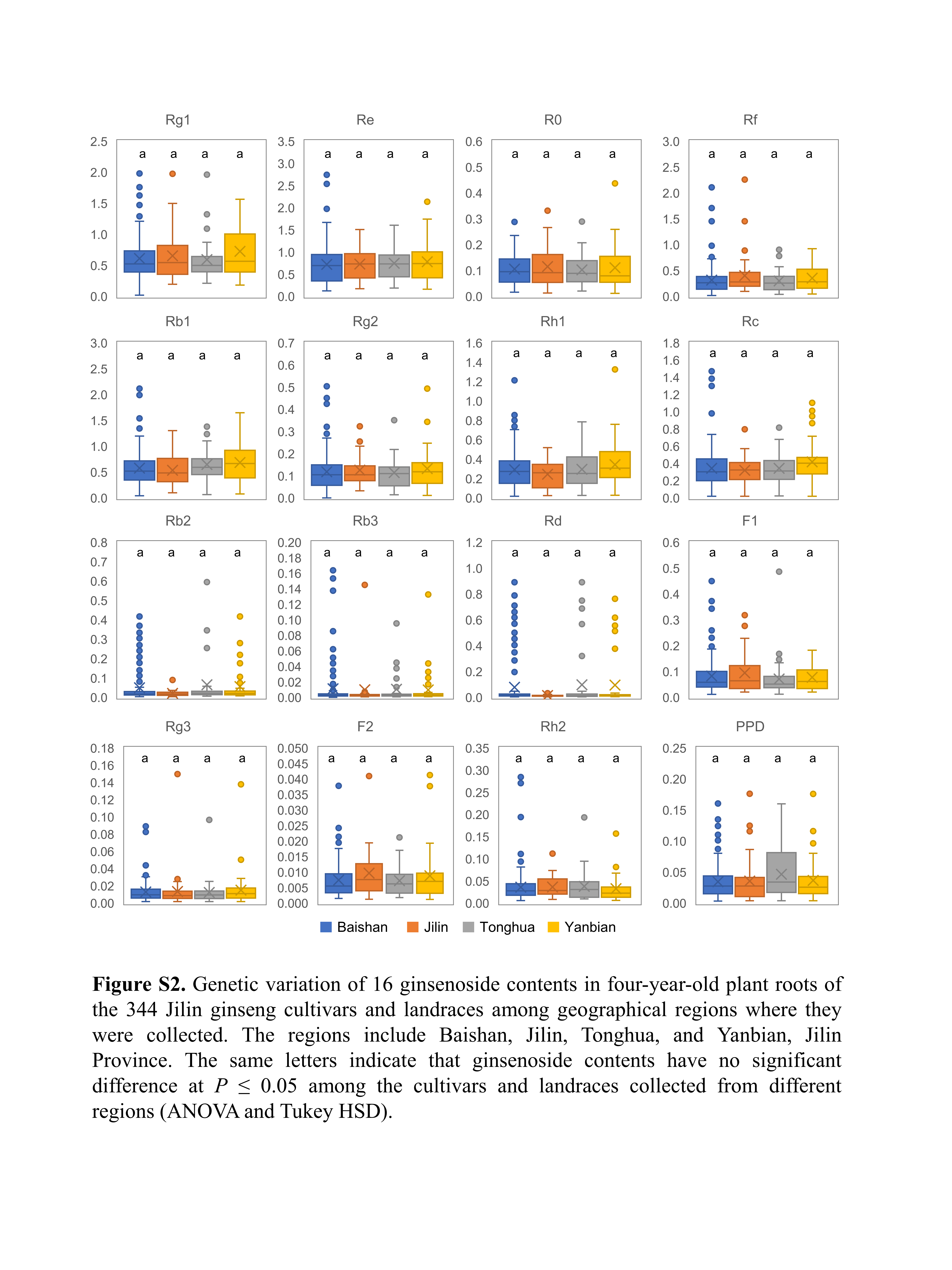

Supplement: Supplementary file 2 [file Image_2.tif]

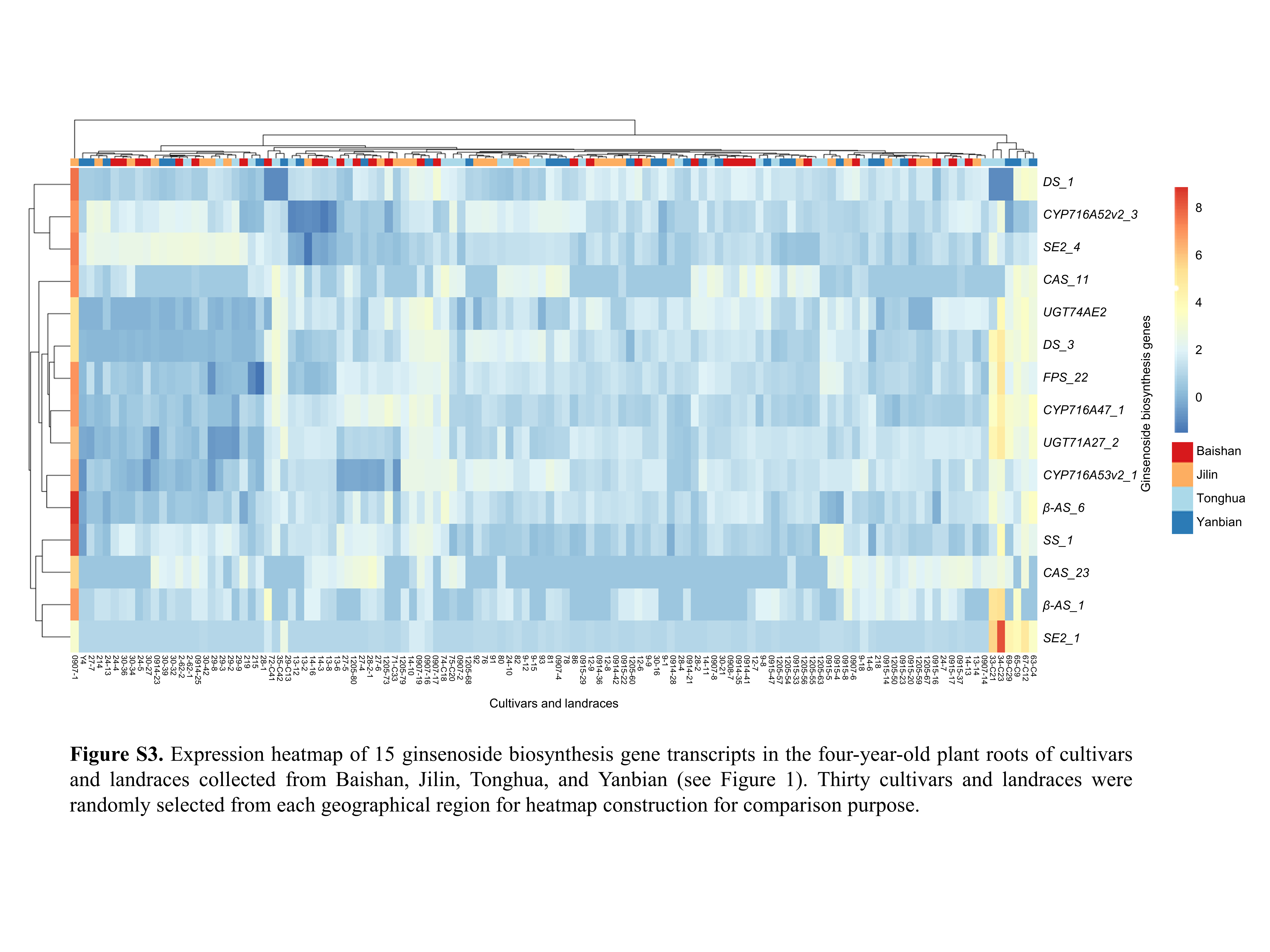

Supplement: Supplementary file 3 [file Image_3.tif]

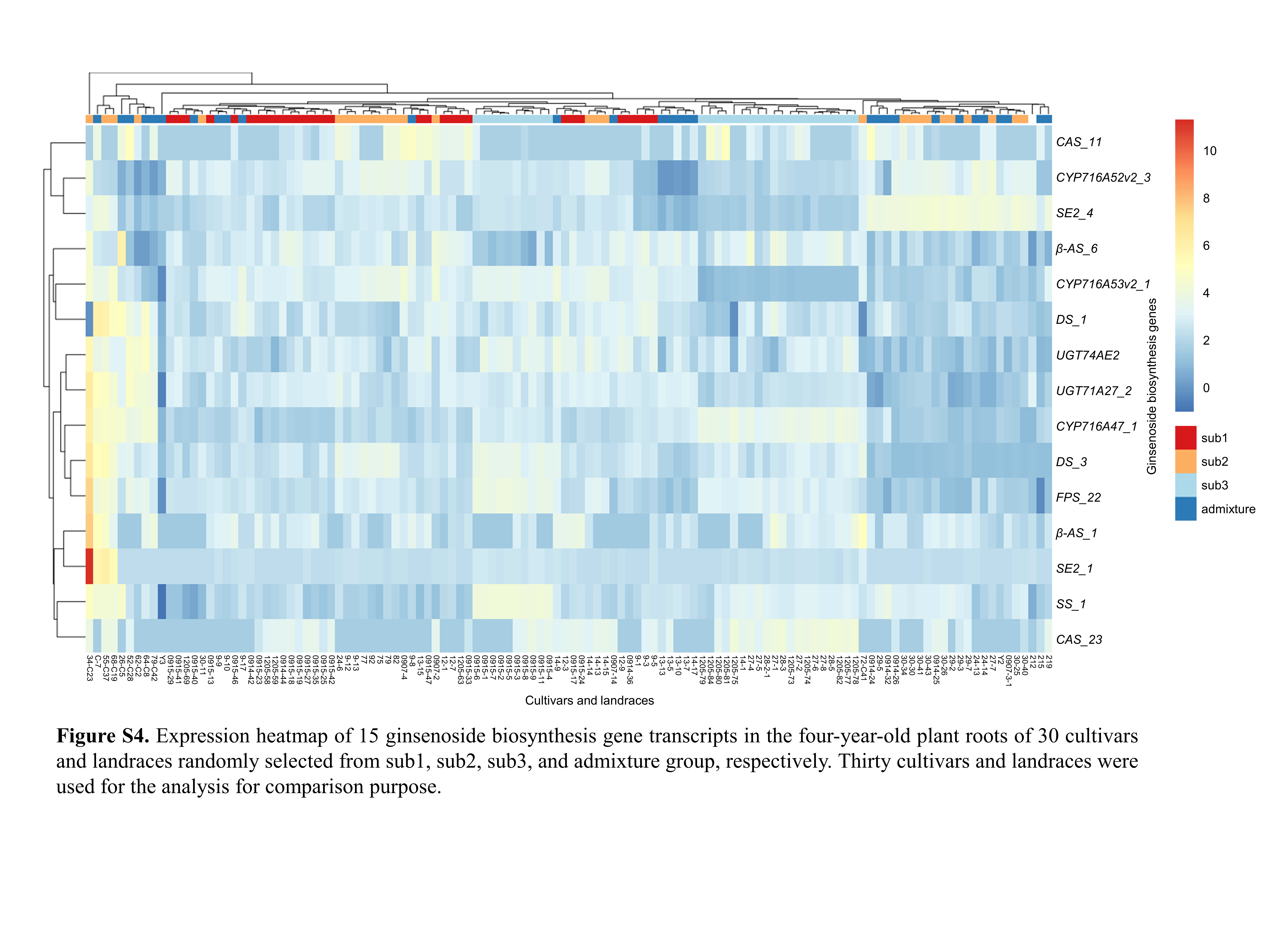

Supplement: Supplementary file 4 [file Image_4.tif]

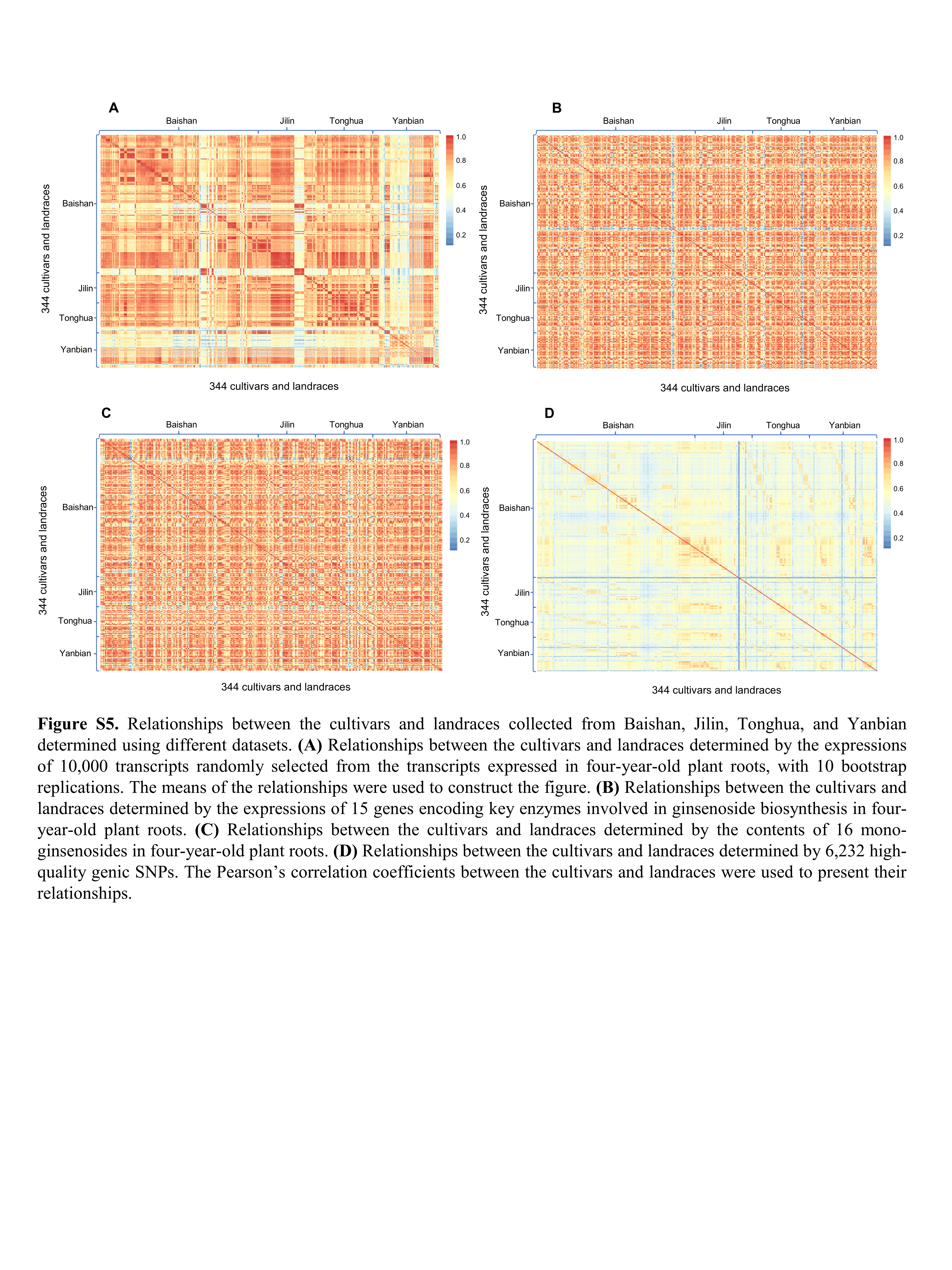

Supplement: Supplementary file 5 [file Image_5.tif]

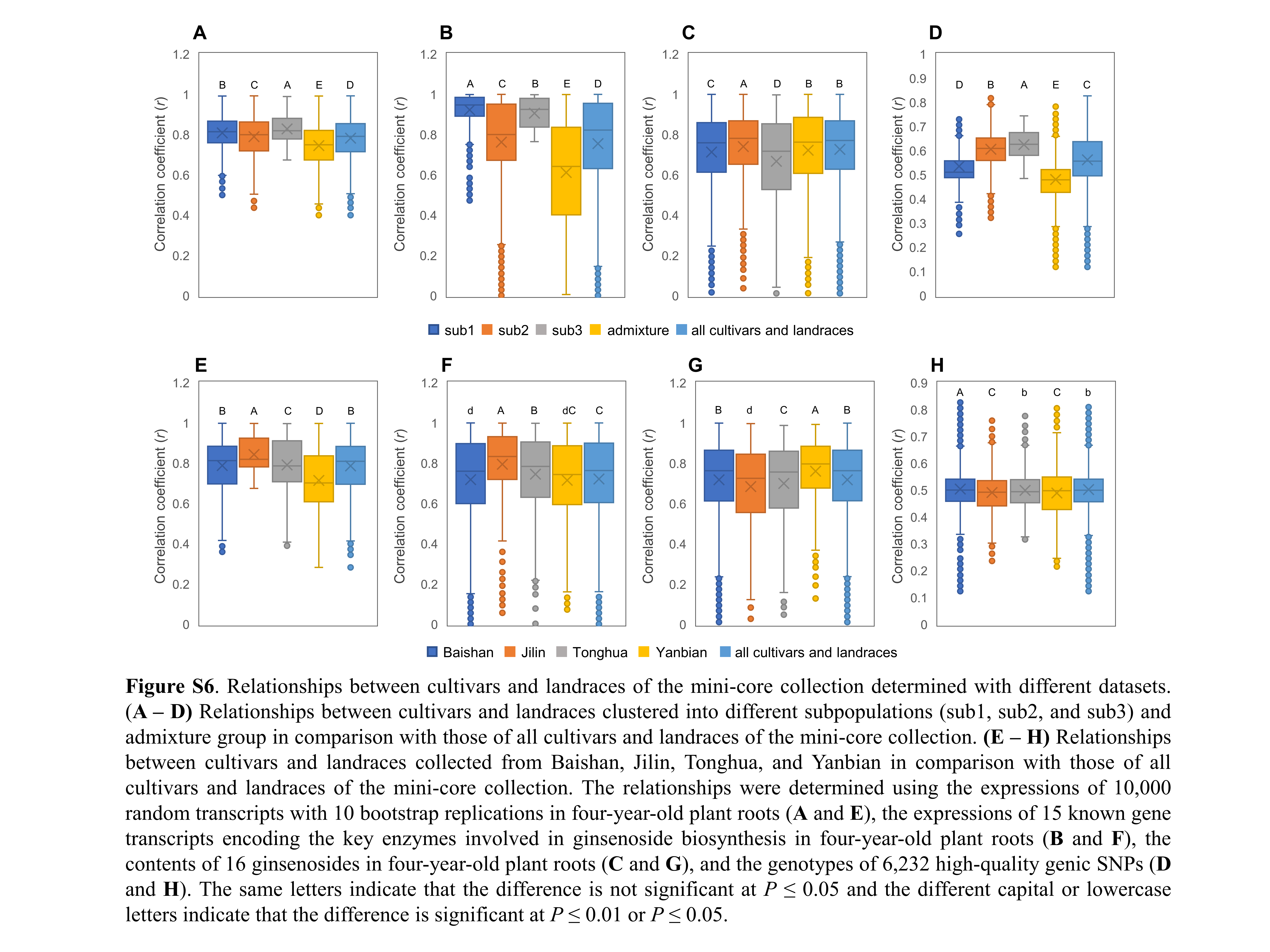

Supplement: Supplementary file 6 [file Image_6.tif]
