# Supplementary material for: Genetic and molecular dissection of ginseng (Panax ginseng Mey.) germplasm using high-density genic SNP markers, secondary metabolites, and gene expressions
Source: Front Plant Sci. 2023 Jul 28;14:1165349. doi: 10.3389/fpls.2023.1165349 (PMC10416250; doi:10.3389/fpls.2023.1165349)
Supplement: Supplementary file 9 [file Table_3.docx]

**Table S3.** Content variation of 16 ginsenosides in the Jilin ginseng mini-core collection. The ginsenosides include Rg1, Re, R0, Rf, Rb1, Rg2, Rh1, Rc, Rb2, Rb3, Rd, F1, Rg3, F2, Rh2, and PPD. The numbers in the parentheses behind the geographical regions indicate the number of cultivars and landraces collected from each of them.

| trait | All (344) | | | Baishan (207) | | | Jinlin (42) | | | Tonghua (42) | | | Yanbian (53) | | |
| --- | --- | --- | --- | --- | --- | --- | --- | --- | --- | --- | --- | --- | --- | --- | --- |
|  | mean | range | CV (%) | mean | range | CV (%) | mean | range | CV (%) | mean | range | CV (%) | mean | range | CV (%) |
| Rg1 | 0.632 | 0.020-1.980 | 55.8 | 0.612 | 0.020-1.980 | 53.4 | 0.654 | 0.195-1.975 | 58.0 | 0.593 | 0.209-1.963 | 57.4 | 0.7256 | 0.180-1.564 | 58.4 |
| Re | 0.733 | 0.123-2.746 | 58.3 | 0.720 | 0.123-2.746 | 62.4 | 0.726 | 0.173-1.509 | 50.0 | 0.745 | 0.187-1.604 | 49.3 | 0.780 | 0.160-2.157 | 56.3 |
| R0 | 0.107 | 0.011-0.437 | 61.0 | 0.106 | 0.016-0.299 | 54.9 | 0.115 | 0.013-0.333 | 70.7 | 0.102 | 0.020-0.289 | 59.8 | 0.110 | 0.010-0.437 | 72.9 |
| Rf | 0.328 | 0.016-2.258 | 88.0 | 0.313 | 0.016-2.107 | 90.8 | 0.400 | 0.097-2.259 | 99.1 | 0.298 | 0.040-0.952 | 72.2 | 0.351 | 0.046-0.923 | 71.0 |
| Rb1 | 0.596 | 0.041-2.199 | 59.2 | 0.575 | 0.041-2.199 | 62.2 | 0.535 | 0.100-1.304 | 55.4 | 0.646 | 0.063-1.407 | 55.0 | 0.686 | 0.078-1.648 | 52.7 |
| Rg2 | 0.121 | 0.000-0.504 | 71.8 | 0.118 | 0.000-0.504 | 73.9 | 0.125 | 0.032-0.339 | 61.9 | 0.114 | 0.014-0.370 | 68.4 | 0.132 | 0.011-0.494 | 74.2 |
| Rh1 | 0.294 | 0.017-1.326 | 67.3 | 0.292 | 0.017-1.214 | 68.0 | 0.244 | 0.024-0.519 | 58.5 | 0.295 | 0.025-0.785 | 62.0 | 0.344 | 0.028-1.326 | 69.0 |
| Rc | 0.353 | 0.019-1.470 | 63.8 | 0.344 | 0.020-1.470 | 67.2 | 0.321 | 0.019-0.800 | 54.7 | 0.344 | 0.023-0.859 | 55.6 | 0.418 | 0.019-1.104 | 60.1 |
| Rb2 | 0.049 | 0.003-0.594 | 177.6 | 0.049 | 0.004-0.435 | 172.1 | 0.021 | 0.003-0.116 | 98.1 | 0.065 | 0.006-0.594 | 185.7 | 0.056 | 0.008-0.417 | 162.9 |
| Rb3 | 0.010 | 0.000-0.165 | 250.7 | 0.011 | 0.000-0.165 | 249.7 | 0.010 | 0.001-0.151 | 319.1 | 0.010 | 0.001-0.098 | 208.8 | 0.010 | 0.001-0.133 | 215.5 |
| Rd | 0.075 | 0.004-0.891 | 236.2 | 0.078 | 0.004-0.89 | 228.4 | 0.014 | 0.004-0.052 | 66.6 | 0.097 | 0.004-0.891 | 226.8 | 0.100 | 0.005-0.762 | 210.7 |
| F1 | 0.082 | 0.012-0.486 | 84.7 | 0.083 | 0.012-0.450 | 85.0 | 0.095 | 0.021-0.318 | 79.9 | 0.071 | 0.012-0.486 | 111.1 | 0.077 | 0.020-0.182 | 65.7 |
| Rg3 | 0.013 | 0.002-0.150 | 120.1 | 0.013 | 0.002-0.089 | 96.2 | 0.014 | 0.002-0.150 | 166.1 | 0.012 | 0.002-0.097 | 128.3 | 0.015 | 0.002-0.138 | 139.4 |
| F2 | 0.008 | 0.001-0.042 | 84.7 | 0.007 | 0.001-0.038 | 80.7 | 0.010 | 0.001-0.042 | 89.7 | 0.007 | 0.002-0.021 | 73.9 | 0.009 | 0.001-0.041 | 91.4 |
| Rh2 | 0.036 | 0.006-0.284 | 91.3 | 0.034 | 0.006-0.284 | 95.3 | 0.036 | 0.008-0.112 | 60.2 | 0.038 | 0.009-0.194 | 93.0 | 0.032 | 0.006-0.157 | 100.7 |
| PPD | 0.036 | 0.003-0.176 | 88.3 | 0.034 | 0.003-0.160 | 81.9 | 0.035 | 0.004-0.176 | 101.5 | 0.046 | 0.004-0.160 | 86.2 | 0.037 | 0.004-0.176 | 96.4 |
