# Supplementary material for: Genetic and molecular dissection of ginseng (Panax ginseng Mey.) germplasm using high-density genic SNP markers, secondary metabolites, and gene expressions
Source: Front Plant Sci. 2023 Jul 28;14:1165349. doi: 10.3389/fpls.2023.1165349 (PMC10416250; doi:10.3389/fpls.2023.1165349)
Supplement: Supplementary file 10 [file Table_4.docx]

**Table S4.** Content **v**ariation of 16 ginsenosides in the Jilin ginseng mini-core collection and its subpopulations and admixture group. The ginsenosides include Rg1, Re, R0, Rf, Rb1, Rg2, Rh1, Rc, Rb2, Rb3, Rd, F1, Rg3, F2, Rh2, and PPD. The numbers in the parentheses behind the subpopulations indicate the number of cultivars and landraces contained in each subpopulation.

| Trait | All (344) | | | Sub1 (80) | | | Sub2 (131) | | | Sub3 (35) | | | Admixture (98) | | |
| --- | --- | --- | --- | --- | --- | --- | --- | --- | --- | --- | --- | --- | --- | --- | --- |
|  | mean | range | CV (%) | mean | range | CV (%) | mean | range | CV (%) | mean | range | CV (%) | mean | range | CV (%) |
| Rg1 | 0.632 | 0.020-1.980 | 55.77 | 0.605 | 0.020-1.980 | 61.25 | 0.625 | 0.020-1.825 | 55.67 | 0.756 | 0.191-1.975 | 61.99 | 0.618 | 0.219-1.627 | 46.16 |
| Re | 0.733 | 0.123-2.746 | 58.33 | 0.742 | 0.173-2.746 | 53.99 | 0.724 | 0.128-1.744 | 53.46 | 0.879 | 0.237-2.745 | 72.67 | 0.685 | 0.123-2.537 | 58.65 |
| R0 | 0.107 | 0.012-0.437 | 60.99 | 0.097 | 0.012-0.299 | 57.41 | 0.123 | 0.013-0.437 | 61.24 | 0.093 | 0.026-0.193 | 51.31 | 0.102 | 0.016-0.332 | 61.70 |
| Rf | 0.328 | 0.016-2.258 | 88.02 | 0.314 | 0.072-1.709 | 76.57 | 0.307 | 0.028-0.952 | 72.29 | 0.459 | 0.102-1.713 | 82.23 | 0.320 | 0.016-2.258 | 110.28 |
| Rb1 | 0.596 | 0.041-2.199 | 59.19 | 0.551 | 0.100-2.113 | 61.73 | 0.597 | 0.042-1.407 | 56.11 | 0.595 | 0.041-2.199 | 82.84 | 0.632 | 0.063-2.033 | 51.87 |
| Rg2 | 0.121 | 0.000-0.504 | 71.82 | 0.123 | 0.024-0.451 | 61.42 | 0.117 | 0.000-0.370 | 71.63 | 0.144 | 0.024-0.504 | 86.93 | 0.116 | 0.000-0.465 | 71.12 |
| Rh1 | 0.294 | 0.017-1.326 | 67.25 | 0.300 | 0.017-0.800 | 61.00 | 0.255 | 0.018-0.759 | 64.79 | 0.327 | 0.029-1.326 | 80.78 | 0.328 | 0.025-1.214 | 65.19 |
| Rc | 0.353 | 0.019-1.470 | 63.79 | 0.322 | 0.019-1.470 | 73.45 | 0.328 | 0.019-1.012 | 60.45 | 0.411 | 0.049-1.406 | 83.47 | 0.390 | 0.031-1.300 | 48.16 |
| Rb2 | 0.049 | 0.003-0.594 | 177.62 | 0.019 | 0.003-0.112 | 74.68 | 0.057 | 0.006-0.594 | 177.9 | 0.018 | 0.006-0.044 | 50.96 | 0.073 | 0.004-0.417 | 141.88 |
| Rb3 | 0.010 | 0.000-0.165 | 250.66 | 0.010 | 0.001-0.153 | 306.72 | 0.012 | 0.001-0.165 | 246.92 | 0.003 | 0.001-0.013 | 91.96 | 0.013 | 0.000-0.155 | 199.27 |
| Rd | 0.075 | 0.004-0.891 | 236.19 | 0.013 | 0.004-0.040 | 58.20 | 0.088 | 0.005-0.891 | 223.14 | 0.017 | 0.005-0.037 | 56.86 | 0.129 | 0.004-0.89 | 176.19 |
| F1 | 0.082 | 0.012-0.486 | 84.70 | 0.076 | 0.012-0.486 | 100.06 | 0.077 | 0.012-0.375 | 78.45 | 0.072 | 0.015-0.343 | 87.83 | 0.101 | 0.020-0.450 | 75.04 |
| Rg3 | 0.013 | 0.002-0.150 | 120.13 | 0.014 | 0.002-0.150 | 139.52 | 0.013 | 0.002-0.080 | 83.47 | 0.013 | 0.002-0.089 | 115.08 | 0.014 | 0.002-0.138 | 137.85 |
| F2 | 0.008 | 0.001-0.042 | 84.73 | 0.007 | 0.001-0.041 | 84.83 | 0.008 | 0.002-0.041 | 79.44 | 0.007 | 0.002-0.023 | 74.25 | 0.009 | 0.001-0.042 | 91.05 |
| Rh2 | 0.036 | 0.006-0.284 | 91.32 | 0.035 | 0.006-0.193 | 89.96 | 0.040 | 0.006-0.284 | 104.34 | 0.028 | 0.009-0.080 | 63.51 | 0.034 | 0.009-0.112 | 62.79 |
| PPD | 0.036 | 0.003-0.176 | 88.31 | 0.043 | 0.004-0.176 | 86.31 | 0.034 | 0.004-0.160 | 92.46 | 0.032 | 0.007-0.125 | 80.97 | 0.034 | 0.003-0.176 | 83.99 |
